# Evaluation of Strategies to Control a Potential Outbreak of Foot-and-Mouth Disease in Sweden

**DOI:** 10.3389/fvets.2017.00118

**Published:** 2017-07-24

**Authors:** Fernanda C. Dórea, Maria Nöremark, Stefan Widgren, Jenny Frössling, Anette Boklund, Tariq Halasa, Karl Ståhl

**Affiliations:** ^1^Department of Disease Control and Epidemiology, National Veterinary Institute (SVA), Uppsala, Sweden; ^2^Department of Diagnostics and Scientific Advice, The National Veterinary Institute, Copenhagen, Denmark

**Keywords:** foot-and-mouth disease, spread model, simulation, vaccination, stamping out, outbreak control

## Abstract

To minimize the potential consequences of an introduction of foot-and-mouth disease (FMD) in Europe, European Union (EU) member states are required to present a contingency plan. This study used a simulation model to study potential outbreak scenarios in Sweden and evaluate the best control strategies. The model was informed by the Swedish livestock structure using herd information from cattle, pig, and small ruminant holdings in the country. The contact structure was based on animal movement data and studies investigating the movements between farms of veterinarians, service trucks, and other farm visitors. All scenarios of outbreak control included depopulation of detected herds, 3 km protection and 10 km surveillance zones, movement tracing, and 3 days national standstill. The effect of availability of surveillance resources, i.e., number of field veterinarians per day, and timeliness of enforcement of interventions, was assessed. With the estimated currently available resources, an FMD outbreak in Sweden is expected to be controlled (i.e., last infected herd detected) within 3 weeks of detection in any evaluated scenario. The density of farms in the area where the epidemic started would have little impact on the time to control the outbreak, but spread in high density areas would require more surveillance resources, compared to areas of lower farm density. The use of vaccination did not result in a reduction in the expected number of infected herds. Preemptive depopulation was able to reduce the number of infected herds in extreme scenarios designed to test a combination of worst-case conditions of virus introduction and spread, but at the cost of doubling the number of herds culled. This likely resulted from a combination of the small outbreaks predicted by the spread model, and the high efficacy of the basic control measures evaluated, under the conditions of the Swedish livestock industry, and considering the assumed control resources available. The results indicate that the duration and extent of FMD outbreaks could be kept limited in Sweden using the EU standard control strategy and a 3 days national standstill.

## Introduction

Foot-and-mouth disease (FMD) is described by the World Organisation for Animal Health (OIE) as “the most contagious disease of mammals” (1). The FMD virus (FMDV, family *Picornaviridae*, genus *Aphthovirus*) causes an acute vesicular disease in cloven-hoofed animals. Seven FMDV serotypes have been described, with cross-protection among serotypes not being observed: O, A, C, Asia1, and SAT1, SAT2, SAT3 (2). Due to its exceptional economic impact, the disease is a high priority in disease surveillance, contingency planning, and trading agreements around the globe. Despite not being a zoonosis, the disease can have severe psychosocial impact for the farming society. The extent of the negative effects of an outbreak in previously free countries, such as economic, social, and in animal welfare, can be demonstrated by the European 2001 outbreak that started in the UK (3).

In 2000 and 2001, outbreaks in the Republic of Korea, Japan, Russia, Mongolia, South Africa, the United Kingdom, Republic of Ireland, France, and the Netherlands were caused by FMDV of serotype O (of a particular genetic lineage named the PanAsia strain) (4). The European outbreak ignited an intense debate regarding the best control strategy during the outbreak, as well as their effect on trading reestablishment after the outbreak. The discussions resulted in a revision of the European Union (EU) legislation for the control of FMD, now established in the Council Directive 2003/85/EC. One of the main new elements of the current legislation, compared to previous ones, is the emphasis on preparation of contingency plans (5). Countries are urged to include the preparation for a “worst-case” scenario in the plan, and contingency plans should be regularly updated in light of current information.

Mathematical modeling was used extensively during the 2001 FMD outbreak, especially in the UK, which was most severely affected (6–10). Since then, it has been a tool for evaluating control strategies in hypothetical scenarios, and supporting decisions when elaborating contingency plans (11–17).

Davis animal disease spread (DADS) is a stochastic simulation model developed at the University of Davis (11) and programmed in R (18). The model was later adapted by the Technical University of Denmark (DTU) to simulate the spread of FMD using different control measures (12, 13, 19). The resulting DTU-DADS model has two main components. *Between-herd* spread is simulated using an agent-based model that simulates FMD spread through direct and indirect contact. *Within*-herd spread is modeled as a compartmental model based on the work of Carpenter et al. (20), and parameterized following (21), as detailed in Ref. (12). Several options for outbreak control have been set up in the DTU-DADS model, which can be enforced in specific herds, buffer zones, or following contact tracing. The model explicitly takes into account the resources available and herds are queued if resources are exceeded.

We used a simulation model to study potential outbreak scenarios in Sweden in case of an introduction of FMD, assess their expected magnitude, and evaluate control strategy options. The model developed is a result of the partnership between epidemiologists from the Swedish National Veterinary Institute (SVA) and the Danish team that developed the spread model DTU-DADS at the Technical University of Denmark (DTU). SVA and the Swedish Board of Agriculture (SJV) worked together to define the main questions to be addressed, and the needed support to the decision-making process of drafting a contingency plan. Emphasis was given to the comparative effect of different control measures.

## Materials and Methods

The DTU-DADS spread model [version 0.15 (19)] was adapted by feeding the model with specific Swedish data, and by adjusting the R codes when needed. All model details are discussed below, and a full description of the model and parameters, including original descriptions from the DTU-DADS model when needed [transcribed from Ref. (12), including updates], are available in the Presentation S1 in Supplementary Material. Model parameterization focused on FMDV serotype O, the same that caused the European outbreaks of 2001, and which is the most widely distributed and prevalent FMDV serotype (4).

**Figure 1:**
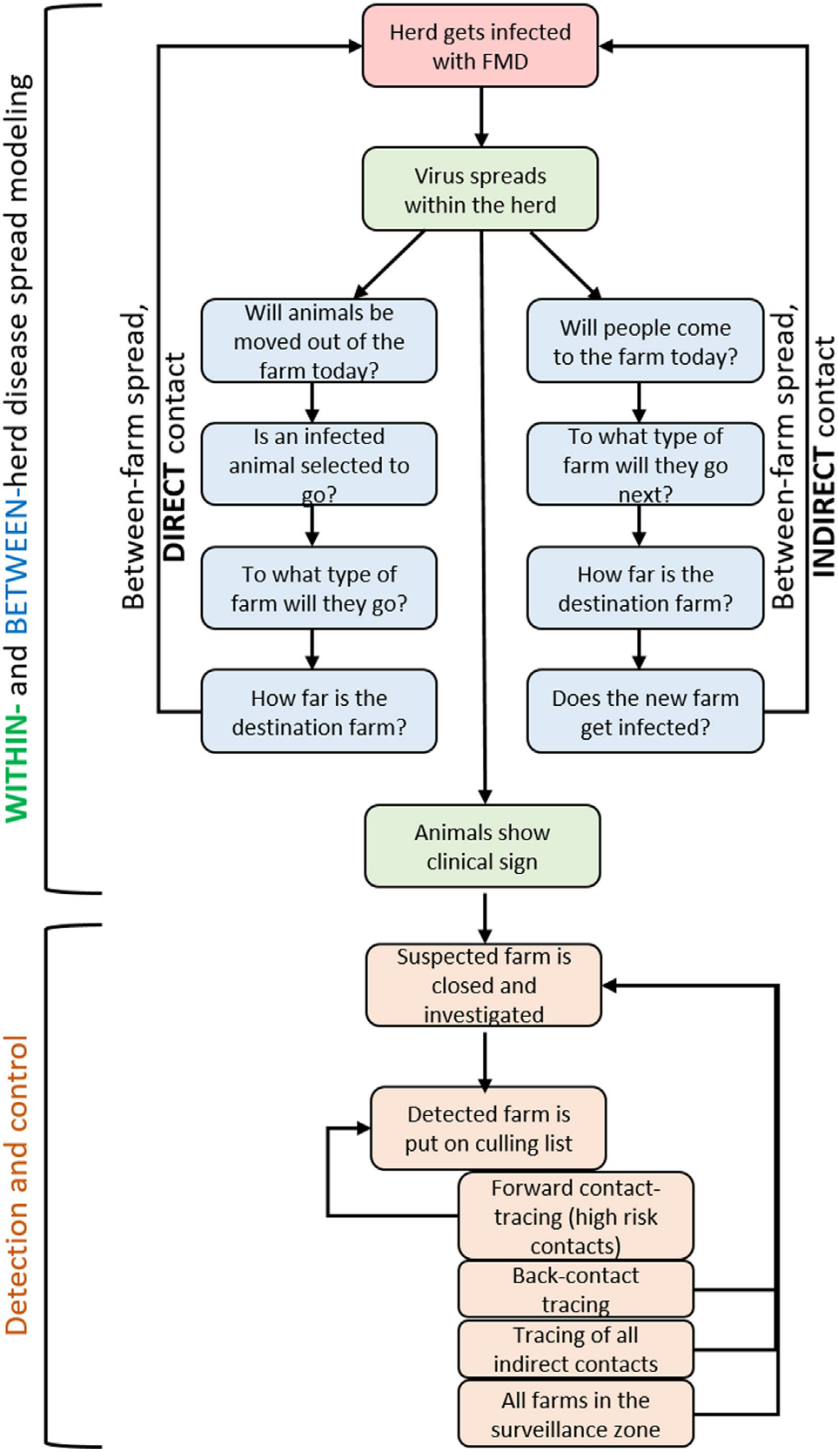
Overview of the events simulated stochastically in the DTU-DADs model.

An overview of the stochastic events simulated in the model is given in Figure 1. Events are simulated in discrete time steps of 1 day. Simulations run from the day of the virus introduction until all infected herds are detected, or up to 365 days if the outbreak is not controlled.

Table 1 lists all model parameters and their sources. Further details for each parameter are given in Presentation S1 in Supplementary Material.

**Table 1:** List of all inputs to the Swedish foot-and-mouth disease (FMD) spread model.

	Parameter	Source	Value^a^
FMD transmission parameters	Probability of transmission when an infected animal is transferred to a new herd	DTU-DADS (12)	Pert (0.95, 0.98, 1)^a^
	Probability of spread by a truck carrying PIGS to slaughter	DTU-DADS (12)	Before detection: Pert (0.005, 0.175, 0.35) After detection: reduction of 20%
	Probability of spread by a truck carrying RUMINANTS (cattle or small ruminants) to slaughter	DTU-DADS (12)	Same as a medium contact risk (see below)
	Probability of spread by a low risk contact (feedstuff trucks, rendering trucks, technicians, visitors, and milk tank trucks)	DTU-DADS (12)	RUMINANTS: Pert (0.005, 0.175, 0.35) PIGS: Pert (*n*, 0.005, 0.1, 0.35)
	Probability of spread by a medium risk contact (persons visiting a farm and expected to visit another farm after, for example, veterinarians, artificial inseminators, and milk controllers)	DTU-DADS (12)	Depends on the herd type. RUMINANTS: Pert (0.1, 0.5, 0.9) PIGS (all specified herd types): Pert (0.05, 0.2, 0.9) PIGS (production type unknown—classified as “others”): Pert (0.1, 0.35, 0.9)
	Local spread	DTU-DADS (12)	95% within 100 m, 1.2% up to 1 km, 0.4% in 2 km, and 0.1% in 3 km
	Disease spread within a herd once an infected animal is introduced	DTU-DADS (12)	Latent period: Poisson distribution with a λ of 3.59, 3.07, and 4.79 for cattle, swine, and sheep, respectively Subclinical period: Poisson distribution with λ of 2.04, 2.27, and 2.16
	Time to detection if clinical signs are present	DTU-DADS (12)	If not detected due to surveillance or tracing, the probability of detection due to clinical signs per day for cattle and pigs: 0.085, 0.17, 0.51, 0.19, 0.06, and 0.07 for days from 1 to 6, respectively. For sheep herds the probabilities for days from 1 to 9 were 0.02, 0.04, 0.27, 0.29, 0.14, 0.12, 0.08, 0.05,5 and 0.12, respectively
Swedish animal population	Cattle herds: (1) geographical coordinates (2) herd size (3) type of herds	CDB database; and Swedish Salmonella surveillance (Estelle Ågren, personal communication)	A total of 23,247 cattle herds were classified into milking (3,427 herds) and not milking. See the Supplementary Material for a map and summary statistics
	Swine herds: (1) geographical coordinates (2) herd size (3) type of herds	Swedish Board of Agriculture database of farms; and personal communication with “Jord På Trynet”	A total of 955 herds were classified into: satellite, weaners, integrated, fattening, KRAV integrated and non-integrated, multipliers, and others. See the Supplementary Material for a map and summary statistics
	Sheep/goats herds: (1) geographical coordinates (2) herd size (3) type of herds	Swedish Board of Agriculture database	A total of 14,885 herds were classified into hobby (15,157) and commercial. See the Supplementary Material for a map and summary statistics
	Probability of sending animals to slaughter	National movement registry (CDB database)	Calculated for each herd individually, per day, based on actual movement data
	Probability of sending animals to other herds	National movement registry (CDB database)	Calculated for each herd individually, per day, based on actual movement data
	Expected distance between herds moving animals and when sending animals to slaughter	National movement registry (CDB database)	Calculated separately for cattle, swine, and sheep, for regions North and South of Sweden, based on actual movement data from 2013. See the Supplementary Material for summary statistics
	Probability of sending animals from each herd type to every other herd type	National movement registry (CDB database)	Calculated separately for cattle, swine, and sheep, for regions North and South of Sweden, based on actual movement data from 2013. See the Supplementary Material for summary statistics
	Average of medium risk contacts coming to the farm, each day	Calculated based on data available from Ref. (22)	Poisson distribution λ= Milking cattle herds: 0.1391881 Non-milking cattle herds: 0.01178143 PIG herds: 0.03535898 SHEEP herds: 0.01377529
	Average of low risk contacts coming to the farm, each day	Calculated based on data available from Ref. (22)	Poisson distribution λ= Milking cattle herds: 0.0904619 Non-milking cattle herds: 0.05215315 Milk tank truck: 0.11 (0.4297846*25%) PIG herds: 0.0821647 SHEEP herds: 0.068445975
	Probability of medium risk contacts going from each herd type to every other herd type	DTU-DADS (12)	“Medium risk contacts from cattle herds were modeled to most often have another cattle herd as the destination herd (88%), while we modeled 60 and 40% of the medium risk contacts to go to other herd types (cattle or sheep) from hobby and non-hobby pig herds, respectively. From sheep herds, we assumed that 50% of the movements were to other sheep herds, while the other 50% were to pig or cattle herds.” Please note that in the Swedish model the herd category equivalent to “hobby” was “others,” which grouped all herds without commercial production type information available
	Distance between farms visited in the same day by low and medium risk contacts	An estimate for Sweden lacks, therefore we used the same distances calculated for the movement of animals
	Distance between the farm and the slaughterhouse	National movement registry (CDB database)	Calculated separately for cattle, swine, and sheep, for regions North and South of Sweden, based on actual movement data from 2013 (CBD database)
	Number of herds visited by a slaughter truck, in average, in one trip to the slaughter house	DTU-DADS (12)	CATTLE: 5 before detection, and 2 after detection PIGS: 1–7, most likely 1 (see specific section for details) SHEEP: modeled as a Poisson distribution with mean 1.5, and 1.2 after detection
	Region	Included in the model by SVA, separating North (listed in the range) from South (all others)	North region includes the following Swedish territories: Värmland, Dalarna, Gävleborg, Västernorrland, Jämtland, Västerbotten, and Norrbotten
Start	Seeding herd	Swedish Board of Agriculture	Chosen depending on the outbreak scenario (see text and Table 2)

Disease control scenarios	Day the epidemic is detected	DTU-DADS (12)	Default values: 21 days Total range evaluated: 17–35 days
	Survey capacity (number of herds that can be visited per day for surveillance)	Swedish Board of Agriculture	40 is the default value, ranges of 20–100 were evaluated
	Culling capacity per day	Swedish Board of Agriculture	Default values: 1,250 ruminants and 2,750 pigs Total range evaluated: 500–3,000 ruminants; 500–3,000 swine
	Vaccination capacity per day	Swedish Board of Agriculture	Default values: 1,000 ruminants and 5,000 pigs Total range evaluated: 500–3,000 ruminants; 3,000–30,000 swine
	Control zones	DTU-DADS (12), and in accordance to EU Council Directive 2003/85/EC	Protection: 3 km from an infected herd Surveillance: 3–10 km
	Number of days to revisit a herd in the surveillance zone	DTU-DADS (12) and 2003/85/EC	14 days
	Delay for the second visit in case a herd is located in the intersection of multiple surveillance zones	DTU-DADS (12) and 2003/85/EC	7 days
	Duration of the surveillance zone	DTU-DADS (12) and 2003/85/EC	30 days
	Movement ban	DTU-DADS (12) and 2003/85/EC	Ban on any animal movement in the country, 3 or 7 days 98% effective [Pert (0.95, 0.98, 1)]
	Time necessary to trace all movements from infected herds	Swedish Board of Agriculture	1 day
	Risk contacts (herds that received animals from infected herds)	DTU-DADS (12) and 2003/85/EC	Compulsory depopulation or put under surveillance only
	Probability of tracing contacts (back and forward) of an infected herd	Swedish Board of Agriculture	98% of tracing and 100% of detecting FMD if the herd is infected
	Probability of tracing indirect, medium risk contacts of an infected herd	Swedish Board of Agriculture	80% of tracing and 99.9% of detecting FMD if the herd is infected
	Probability of tracing indirect, low risk contacts of an infected herd	Swedish Board of Agriculture	50% of tracing and 99.9% of detecting FMD if the herd is infected
	Ring depopulation	DTU-DADS (12) and 2003/85/EC	Radius: 500, 1,000, 1,500 m Enforced: 1 or 14 days after detection of the epidemic; or enforced after 10, 20, or 30 herds are detected
	Ring vaccination	DTU-DADS (12) and 2003/85/EC	Radius: 1,000, 2,000, 3,000 m Enforced: 7 or 14 days after detection of the epidemic; or enforced after 10, 20, or 30 herds are detected
	Vaccination efficiency	DTU-DADS (12)	Pert (0.39, 0.42, 0.47)
	Vaccination immunity built up	DTU-DADS (12)	See appropriate section for detailed number per day
	Behavior changes after detection	Swedish National Veterinary Institute (SVA)	Medium risk contacts would reduce with a probability Pert (0.7, 0.8, 0.95) and low risk contacts Pert (0.2,0.3,0.5)

*Further details for each parameter are given in the Supplementary Material*.

*^a^Probability distribution used (minimum, most likely, and maximum)*.

All disease transmission parameters that were thought to be readily applicable from the Danish to the Swedish livestock population, or to be independent from the host population (intrinsic pathogen properties) were kept as set up in the DTU-DADS model, as explained individually for the parameters in Table 1 and Presentation S1 in Supplementary Material.

To adapt the model to the Swedish livestock population, specific data were collected for all FMD susceptible herds in Sweden, including animal movement data, as shown in Table 1 and detailed in Presentation S1 in Supplementary Material. The direct and indirect contact networks among these herds were also characterized. Animal and people movements were characterized and modeled according to herd type, but independently for two main geographical regions in Sweden: North and South. This was to account for the lower farm density in the north of Sweden.

The model considers each group of animals from the same species, within the same farm, as one herd, and models herds individually; if a farm contains cattle and pigs, for example, cattle and pig herds are modeled individually. A farm ID is used to keep track of herds in the same farm, and enforce control measures in all herds within a farm equally. If for instance one of the herds is detected as infected, all herds belonging to the same farm are culled. A high probability of local area spread within 100 m is used to account for horizontal transmission between herds in the same farm.

Outbreaks were modeled under different scenarios of disease introduction, to assess the effect of different population parameters in the development of the outbreak. In each of 21 base scenarios, outbreaks were set to start in a herd of a particular type, and in each iteration, the first infected herd was randomly selected among all herds of that type. Seven scenarios had infection seeded in the south of Sweden, in one specific herd type (dairy cattle, cattle herds without milking activity, sow herds, fattening pig herds, weaners, multiplying pig herds, or small ruminant herds); another seven scenarios were related to the same herd types, but seeded in the north of Sweden; and finally herds were chosen based on the frequency of direct animal contacts in a year (low, medium or high contact network cattle herds; low, medium or high contact network pig herds; or high contact network small ruminant herds). In addition, spread was also evaluated when 2, 3, or 4 initial seeds were set (number of infected herds to start the epidemic), all in cattle herds. The evaluated scenarios are listed in Table 2.

**Table 2:** List of all evaluated scenarios of disease spread and control measures.

	Disease seeding/spread settings			# of Seed herds	Disease control settings
	Species	Herd characteristics	Region		
Base control scenarios	Cattle	Milking	South	1	Base control strategy: Three days ban of all susceptible livestock movements after first detection (standstill) Establishing of a 3 km protection zone and a 10 km surveillance zone around every detected herd, with all herds within zones visited for clinical inspection Culling of all animals in detected positive farms and their high risk contacts (farms that received animals from the infected ones)
		Not milking			
	Pigs	Sows			
		Fattening			
		Weaners			
		Multipliers			
	Small ruminants	Any			
	Cattle	Milking	North	1	
		Not milking			
	Pigs	Sows			
		Fattening			
		Weaners			
		Multipliers			
	Small ruminants	Any			
	Cattle	Low trade frequency	Both regions	1	
		Medium trade frequency			
		High trade frequency			
	Pigs	Low trade frequency			
		Medium trade frequency			
		High trade frequency			
	Small ruminants	High trade frequency			
	Cattle	All herds	South	2	
	Cattle			3	
	Cattle			4	

Effect of alternative control measures	Cattle	Medium trade frequency	Both regions	1	Earlier detection (days 17–21) Late detection (days 21–25) Lowered surveillance resources (20 herd visits/day) Increased surveillance resources (100 herd visits/day) Standstill increased to 7 days Standstill of 3 days, but NO reduction in the movement of people after detection Depopulation in a radius of 1,000 m around every infected farm, enforced 1 day after detection of the outbreak Culling capacity decreased to 500 ruminants and 500 swine per day Culling capacity increased to 3,000 ruminants and 3,000 swine per day Depopulation in a radius of 500 m, enforced 1 day after detection of the outbreak Depopulation in a radius of 1,500 m, enforced 1 day after detection of the outbreak Depopulation in a radius of 1,000 m, enforced 7 days after detection of the outbreak Depopulation in a radius of 1,000 m, after 14 days Depopulation in a radius of 1,000 m, enforced after 10 detected herds are detected Depopulation in a radius of 1,000 m, 20 detected herds Depopulation in a radius of 1,000 m, 30 detected herds Vaccination in a radius of 1,000 m around every infected farm, enforced 1 day after outbreak detection. All vaccinated animals culled at the end Same as previous scenario, but vaccination capacity reduced to 500 ruminants and 3,000 swine per day Vaccination capacity increased to 3,000 ruminants and 30,000 swine per day Vaccination in a radius of 2,000 m, enforced 1 day after outbreak detection. All vaccinated animals culled at the end Vaccination in a radius of 3,000 m, enforced 1 day after outbreak detection. All vaccinated animals culled at the end Vaccination in a radius of 1,000 m around every infected farm, enforced 7 days after outbreak detected Vaccination in a radius of 1,000 m around every infected farm, enforced 14 days after outbreak detected Vaccination in a radius of 1,000 m around every infected farm, enforced after 10 detected herds Vaccination in a radius of 1,000 m around every infected farm, enforced after 20 detected herds Vaccination in a radius of 1,000 m around every infected farm, enforced after 30 detected herds

Sensitivity analysis using worst-case scenarios	Tested for each of 3 “worst-case scenarios”: (A) Cattle milking herd, any region (B) Pig herd with high trading frequency (C) 4 seed cattle herds in the South of Sweden (any herd type)				Capacity of only 20 surveillance visits per day Limited culling resources (500 ruminants and 500 swine) Effectiveness of standstill (prohibition of moving animals in the whole country for 3 days)—reduction of 15% and then 40% on the originally set effectiveness Effect of detection on the behavior of people (reduction in indirect contacts between farms)—reduction of 15% and then 40% on the originally set effectiveness Time to trace contacts increased to 3 days Probability of tracing direct contacts—reduction of 15% and then 40% on the originally set effectiveness Probability of tracing indirect contacts—reduction of 15% and then 40% on the originally set effectiveness Probability of detecting traced contacts if indeed positive—reduction of 15% and then 40% on the originally set effectiveness Effectiveness of enforcing control measures in the detected farms—reduction of 15% and then 40% on the originally set effectiveness Effectiveness of enforcing control measures in the surveillance zone—reduction of 15% and then 40% on the originally set effectiveness Late detection: 28 and 35 days

Chaos scenarios	Cattle	All herds	South	4	Base control strategy Detection only on day 28 after introduction All the control measures listed in the sensitivity analysis above with effectiveness reduced by 15% compared to the values considered realistic for Sweden Surveillance capacity and culling capacity were kept normal (40 surveillance teams, culling capacity of 1,500 ruminants, and 3,300 swine)
					Base control strategy + detection on day 28 + effectiveness of control measures reduced by 15% + normal surveillance capacity + Increase standstill to 7 days
					Base control strategy + detection on day 28 + effectiveness of control measures reduced by 15% + normal surveillance capacity + Culling of all animals in a radius of 1 km around every infected farm, enforced as soon as 10 herds were detected
					Base control strategy + detection on day 28 + effectiveness of control measures reduced by 15% + normal surveillance capacity + Vaccination of all animals in a radius of 3 km around every infected farm, enforced as soon as 10 herds were detected

*One-thousand iterations were modeled for each scenario*.

Base scenarios were simulated using a fixed control strategy (here we use “control strategy” to denote a specified collection of “control measures”). In these *base control scenarios* the mandatory conditions determined in the EU Council Directive 2003/85/EC were implemented, and in addition a 3-day national standstill:

- Culling of all animals in detected FMD-positive farms and their high risk contacts (farms that received animals from the infected ones).
- Establishing of a 3 km protection zone and a 10 km surveillance zone around every detected farm. Susceptible animals’ movement prohibition is kept in these zones for 30 days, a period during which all herds are visited for clinical inspection twice, starting from the protection zone. More details are given in Presentation S1 in Supplementary Material (Section 1.4.2 in Supplementary Material).
- 3-day national standstill, i.e., ban of all susceptible animal movements after first detection.

A reduction in the number of indirect contacts among farms after detection of the outbreak was also enforced, as per parameters listed in Table 1. The standard detection day used in the DTU-DADS model (21 days) was set, and the estimated surveillance capacity in Sweden is listed in Table 1.

After the effects of different scenarios of disease introduction were evaluated with this base control strategy, one of the *base control scenarios* was chosen to evaluate the effect of applying alternative control strategies. The choice was based on the evaluation of the *base control scenarios* and is described in the results. *Alternative disease control measures* were evaluated, using a range of parameters listed in Table 1 (see Table 2 for a list of the evaluated scenarios):

- Preemptive depopulation (ring culling), for different ring radii, and triggered when a determined number of detected infected herds was reached, or outbreak control was not reached after a number of days;
- Ring vaccination (vaccination to cull), using different ring radii, and also dependent on the number of detected infected herds reached, or the outbreak length.

Based on the results of previous scenarios, three *worst-case scenarios* were chosen. *Sensitivity analysis* was carried out in these worst-case scenarios to ensure that the effect of different parameters could be more easily identified. Model sensitivity was evaluated against a range of values for the detection day and the effectiveness of the alternative control measures, and variation in the amount of surveillance resources available (daily survey and culling capacity, see Tables 1 and 2).

Finally, to confirm the conclusions drawn from the previous steps, all disease spread conditions determined to have high impact in the outbreak size were manipulated to exaggerate the worst-case scenarios, and create a *chaos scenario*. “Chaos” was assumed to be a consequence of a very high infection pressure to start with (four infected herds to start the epidemic, all in the south of Sweden and close to the Danish border), and a cumulative number of failures in the effectiveness of all control measures applied. These conditions were intended to mimic an epidemic that starts and develops with a much greater magnitude than expected, compared to the typical outbreak scenarios modeled previously, or an epidemic that gets out of control. The effectiveness of specific control measures were challenged against this chaotic scenario (see Table 2).

Table 2 lists every scenario evaluated. For each scenario, the following outputs are reported:

- Epidemic duration, in days, defined as the day from first detection until the day when the last infected herd was detected (note that control would then still continue until all infected farms are culled and surveillance and protection zones are lifted);
- Total number of herds infected during the course of the epidemic;
- Total number of herds visited by surveillance teams (herds put in surveillance queue due to being direct or indirect contacts of an infected farm, or for being in a surveillance zone);
- Total number of herds culled, and total number of animal in these herds;
- Total number of herds vaccinated, and total number of animals in these herds.

Ten-thousand iterations of scenario 1 showed that output medians and interquartile ranges were stable after 500 iterations, but the maximum varied due to longer epidemics observed in individual iterations when more repetitions were run. As a compromise between achieving higher variability and keeping computational time manageable, 1,000 iterations were simulated for each scenario.

The progression of scenarios described above focused on testing the model sensitivity to the control measures. To also evaluate the structure of the model, and the impact of the parameters that were imported from the Danish model, the transmission parameters listed in Table 1 were also subjected to sensitivity analysis. The probability of transmission associated with direct contact, slaughter trucks, low risk contact, and medium risk contact were increased and reduced to up to 20%. The effect of local spread was also subjected to sensitivity analysis, by removing any local spread that was not between herds in the same farm, or increasing the probabilities in radius from 1 to 3 km up to five times.

## Results

In general, the results showed that an FMD outbreak in Sweden would most likely be small and of short duration, and that base control measures as specified in the EU legislation, complemented with a 3-day national standstill of all susceptible animals movements, would be enough for bringing the outbreak under control. Considering the 24 base scenarios evaluated, the median epidemic duration (time from detection of the first infected herd to the day in which the last herd was detected) was 3–15 days, and the median number of infected herds was 2–19 (with a median number of culled animals of 46–4,136). The 95% percentiles were for an epidemic of 20 days, involving 15 infected herds and culling nearly 5,000 animals.

Summary statistics for all the scenarios evaluated are presented in Table S2-1 in Presentation S2 in Supplementary Material and Table 1, and relevant results and conclusions are presented and discussed by group of scenarios below. Please note that epidemic duration is counted from the detection day. Simulations in which the epidemic was considered to die off before detection resulted in negative epidemic duration.

**Figure 2:**
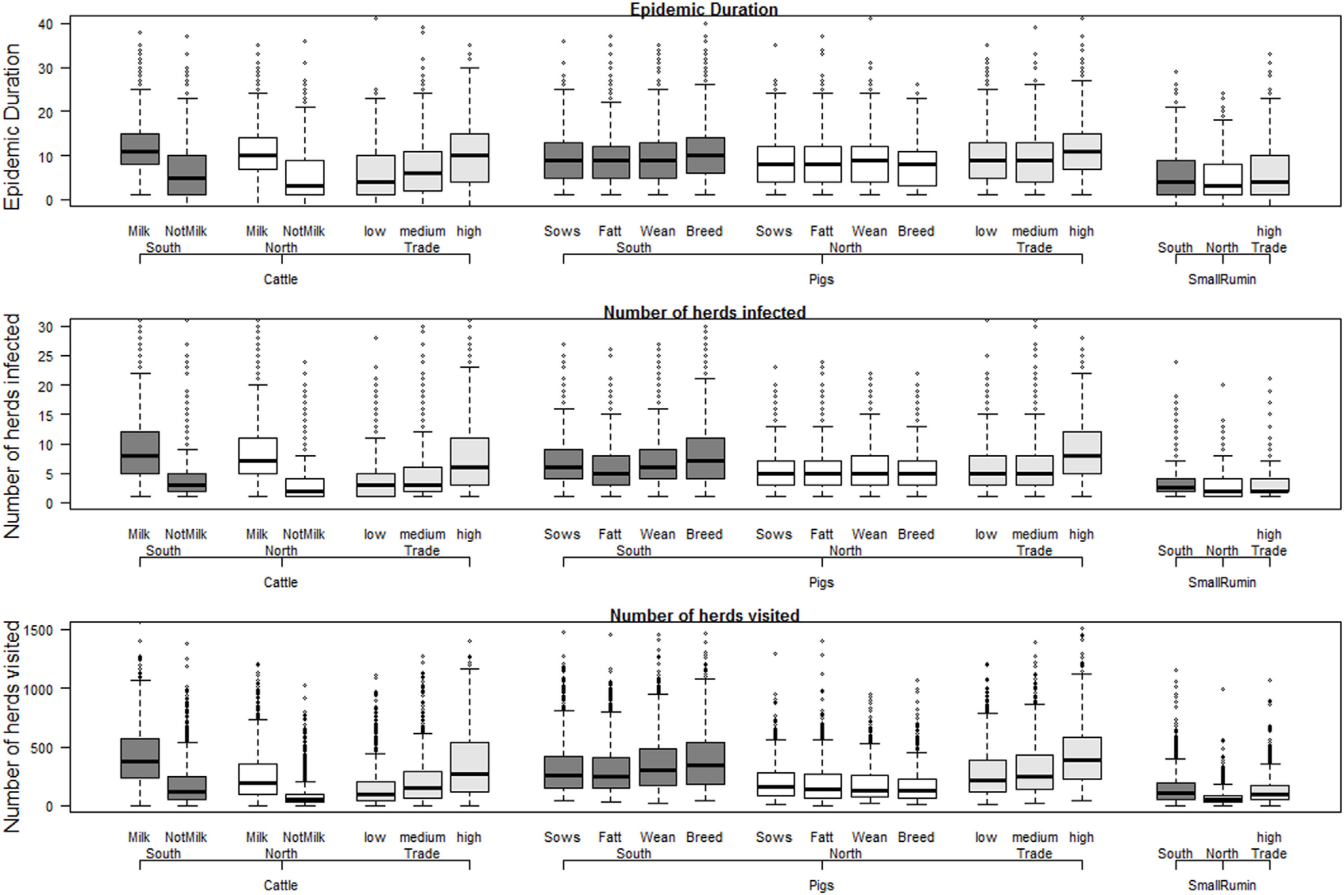
Results for the base scenarios with outbreaks starting in the South, North, or based on number of contacts (trade), per herd type and herd species. Individual box plots represent the summary of 1,000 iterations for each scenario. All scenarios are detailed in Table 2, but in short: Milk = cattle herds delivering milk; NotMilk = herds without any reported milking activity; Sows = sow pools; Fatt = fattening pig herds; Wean = weaners pig herds; Breed = multiplier pig herds; SmallRum = small ruminant herds (sheep, goats, or both).

The results of the base scenarios (Figure 2) showed that the region where the outbreak started (North versus South) had little effect on the expected size and duration of the epidemic. By looking in detail into individual iterations, and mapping every modeled transmission event, it was possible to conclude that this was because epidemics starting in the North eventually spread to the South through long distance movements. The main difference between epidemics starting in the North and South are the resources needed to control the outbreak, as farm density is lower in the North, and therefore a smaller number of farms ends up in the surveillance zones. The median number of farms that needed to be visited by surveillance teams (direct contacts of infected farms, or farms within the surveillance zones) was 106–381 for the base control scenarios in which epidemic started in the South (95% percentile = 448–915) and 49–195 in the North (95% = 188–734).

The effect of species and herd types seemed to be a direct effect of the contact network structure for each herd type. Epidemics starting in sheep/goats herds were generally smaller, due to a lower probability of direct (animal movement) and indirect (people movement) contacts. Cattle herds had very different results depending on whether it was a dairy herd or not, reflecting the larger number of indirect contacts expected daily in herds with milking animals. Outbreaks starting in pig herds in general resulted in an average epidemic size between milking and non-milking cattle herds. The main impact of starting epidemics in pig herds was the higher number of animals that were culled, a reflection of their much larger herd size (see Presentation S1 in Supplementary Material for herd statistics). As epidemics were generally small, with only a few herds being culled, the size of the seeding herd had high impact on the total number of culled animals. Only the number of animals culled is shown in Figure 2, since the number of herds culled was almost always the same as the number of infected herds (Table S2-1 in Supplementary Material).

The number of pig herds in Sweden is very small, and as a result, epidemics that started in pig herds were ultimately driven by spread among cattle herds, as we could conclude from extensive analysis of the base control scenarios. Since cattle herds seemed to be driving spread, and the contact network (direct and indirect contacts) was the main driver of the epidemic size, a “typical outbreak scenario” was chosen as one starting in a cattle herd in the south of Sweden, with an average number of yearly direct contacts. This scenario was chosen to test the effect of alternative control measures, as shown in Table 2.

**Figure 3:**
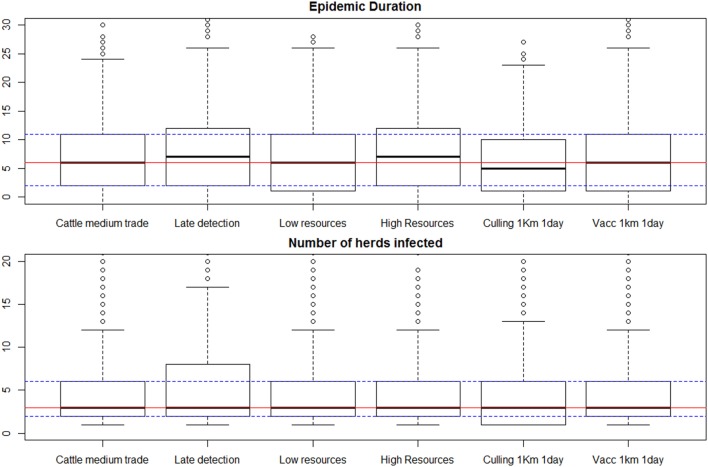
Results of selected scenarios comparing alternative control measures and amount of resources available. Scenario labels are as presented in Table 2. Individual box plots represent the summary of 1,000 iterations for each scenario. Red lines mark the median for all the iterations in the “typical outbreak scenario” against which all measures are compared (first box plot), and the dashed lines represent the 25 and 75% percentiles for that scenario.

The main result for all scenarios designed to evaluate the effect of alternative control measures (listed in Table 2) was a remarkable lack of variation between these scenarios, as demonstrated for a few selected scenarios in Figure 3, and for all scenarios in Presentation S2 in Supplementary Material (Figure S2-1 in Supplementary Material). Late detection (modeled as a pert distribution from 21 to 25 days, with most likely 23 days) had an effect in increasing the epidemic duration and the maximum observed number of infected herds, but not increasing the median number of infected herds. In all the different scenarios simulated the median number of infected herds was 3, and the 95% percentile ranged from 12 to 15 for all scenarios but late detection, in which the 95% percentile for the number of infected herds was 19. Ring culling had only a marginal effect in reducing the epidemic duration, but not the number of herds infected. Ring vaccination did not reduce the epidemic duration nor the number of herds infected.

The “typical outbreak scenario” was examined on a daily basis, focusing on the number of herds put in the surveillance list daily, in comparison to the number of available teams. The results (Figure S2-3 in Supplementary Material, Presentation 2) showed that the number of herds to be visited per day only exceeded the capacity of surveillance immediately after detection in the median outbreak, with herds waiting at most a day to be visited in the cases when the outbreak was controlled within a week. Epidemics in iterations placed above the 90% percentile could take up to 19 days to be controlled. In those cases the number of days a herd would have to wait to be visited could be as high as 14, with daily medians ranging from 0 to 8.

Sensitivity analysis were performed using three “worst-case scenarios” to magnify the observed effectiveness of control measures, which could have been hard to observe in the small outbreak sizes associated with the “typical outbreak scenario.” The sensitivity analysis showed that these results were robust for the range of parameters tested in the sensitivity analysis (see sensitivity analysis section in Table 2), except for one: the day of detection (Figure S2-2 in Supplementary Material, Presentation 2). Table 2 lists the 19 scenarios evaluated based on worst-case scenario A (cattle scenario with highest expected epidemic size and duration—starting in a milking herd). If we exclude the two scenarios in which late detection was tested, the median number of infected herds for all other 17 scenarios ranged from 7 to 8, and the median epidemic duration was always 11 days (from detection of the first until detection of the last infected herd). Each week of delayed detection doubled the median number of infected herds, resulting in medians 16 and 32 herds for the scenarios of detection on days 28 and 35, respectively. The median epidemic duration for the late detection scenarios were 14 and 18 days.

For worst-case scenario B (starting in a pig herd with a great number of direct contacts), the median number of infected herds in the 17 scenarios tested with detection on day 21 (but varying the efficacy of various control measures) ranged 10–11, and the median epidemic duration was always 13 days. Detection on days 28 and 35 increased the median number of infected herds to 30 and 61, respectively, and resulted in a median epidemic duration of 17 and 21 days.

When the epidemic was seeded in four cattle herds at the same time (worst-case scenario C), but detection was not delayed, the median number of infected herds in all sensitivity analysis scenarios evaluated varied between 19 and 20 herds, with median epidemic duration varying between 15 and 16 days. Detection on days 28 and 35 increased the median number of infected herds to 40 and 87, respectively, and resulted in a median epidemic duration of 19 and 24 days.

Sensitivity analysis for the transmission parameters showed that the results were very robust to changes in punctual transmission parameters. As for the previous analysis, this was particularly true in scenarios with low expected number of infected herds. In the “typical outbreak scenario,” for instance, changes of up to 20% in the probability of transmission following direct contact did not change the median number of infected herds. Evaluation of the percentage of all transmission events, over all iterations in that scenario, showed that about 45% were a result of direct contact, and 5% of movement to slaughter. This resulted in robustness of the model to changes in the probabilities of transmission associated with slaughter movements. About 28% of the transmission events were due to indirect contact (low and medium risk contacts), and 22% due to local spread. The probability of local spread within 100 m was kept high in all scenarios to ensure transmission between herds within the same farm. As expected, increases in the probability of transmission for other distances resulted in a higher number of infected herds, but a fivefold increase in the probability of transmission within 1 km, for instance, only increased the median number of infected herds in the typical scenario by about two herds.

**Figure 4:**
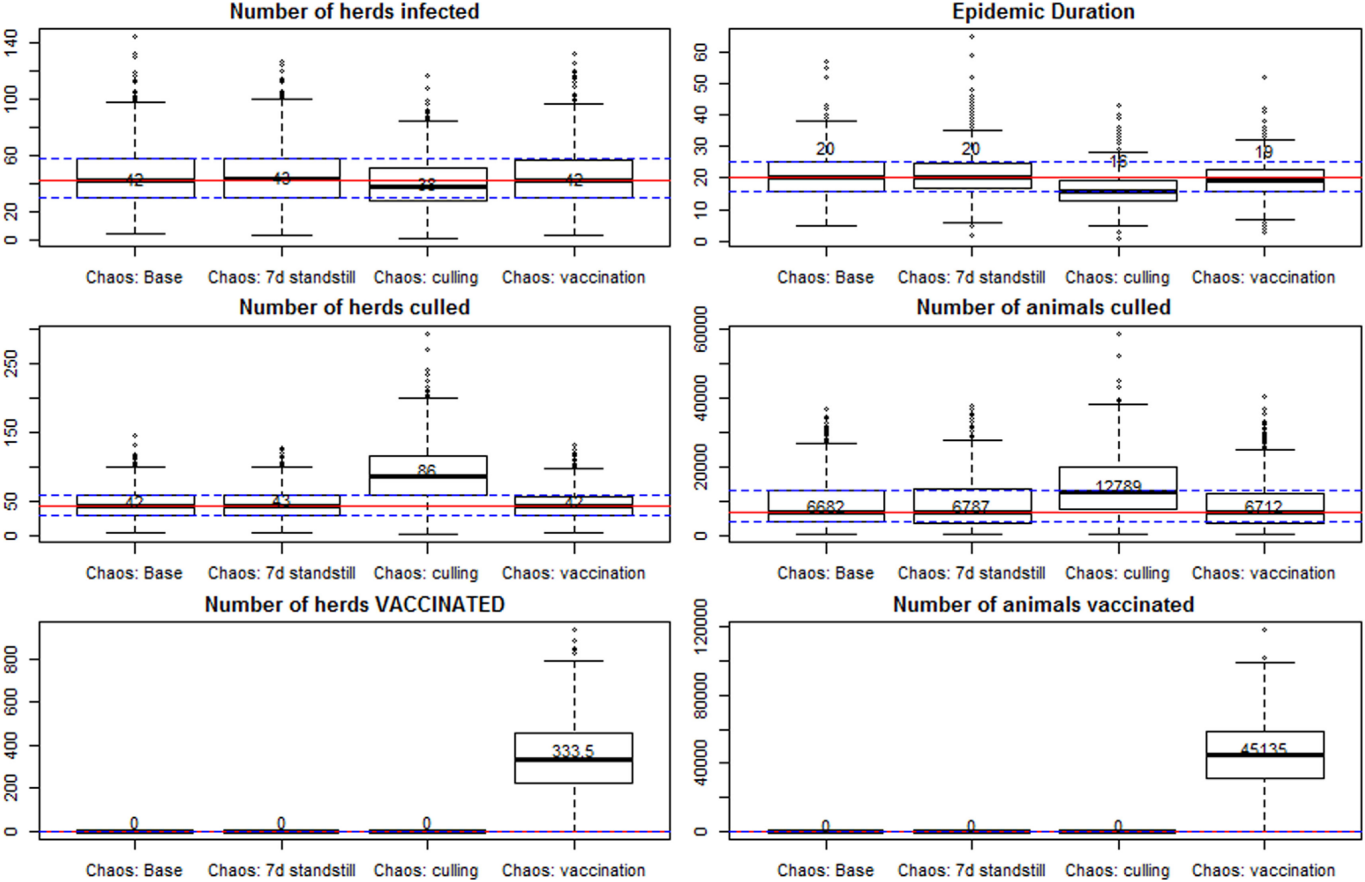
Results of spread under a scenario of “chaos,” with only base control measures in place, and with implementation of additional controls. All scenarios are further detailed in Table 2. Individual box plots represent the summary of 1,000 iterations for each scenario. Red lines mark the median for all the iterations in the scenario with base control measures (first box plot), and the dashed lines represent the 25 and 75% percentiles for that scenario.

Based on the results of scenarios presented above, a cutoff of 10 detected infected herds was set as a decision point for when authorities should start considering that the outbreak was not being brought under control. In all base scenarios the expected number of infected herds was under 10, and only higher in scenarios with multiple starting seeds or failures in the effectiveness of control measures. The effect of deciding to implement ring culling or vaccination after this threshold was reached was evaluated in the chaos scenarios, and results are presented in Figure 4.

The scenario with infection seeded in four cattle herds in the south of Sweden at the same time, and detection after 4 weeks (base chaos scenario), resulted in a median number of 42 infected herds (95% percentile of 83 herds), and an epidemic duration of 20 days between detection of the first and last herd (95% percentile at 33 days). This is assuming that all base control measures would be applied, and the surveillance capacity would be at a regular level, but all applied control measures would be 15% less effective than in the base scenarios (for instance effectiveness of enforcement of the standstill, and effectiveness of tracing). Increasing the period of standstill was not effective in reducing the number of infected herds nor the epidemic duration. Ring vaccination was not effective in reducing the median number of herds infected, although the median epidemic duration was reduced by 1 day (median 19 and 95% percentile of 28 days).

The implementation of preemptive depopulation of all susceptible animals, in a radius of 1 km around every infected farm, would reduce the median epidemic duration in the chaos scenarios by 4 days (median 16 days; 95% percentile at 26 days). The median number of infected herds was reduced to 38 (95% percentile at 72 herds). As a consequence of the reduction in the number of infected herds (fewer surveillance zones to be established), the median number of visited herds was reduced from 1,075 in the base chaos scenario to 875 when culling was applied (95% percentiles at 1,439 and 1,327, respectively). The median number of culled herds, however, was increased from 42 to 86 (95% percentiles at 82 and 179, respectively), and the median number of animals culled from 6,682 to 12,789 (95% percentiles at 23,002 and 29,897, respectively, for the scenarios without and with preemptive depopulation).

## Discussion

A disease spread model was adapted to the Swedish livestock structure to evaluate the effect of different control strategies and inform FMD preparedness in Sweden. Results showed that an FMD introduction in Sweden will most likely spread slowly and be readily contained with adoption of a control strategy combining the control measures required in the current EU legislation for the control of FMD (Council Directive 2003/85/EC), and a national standstill. The detailed control strategy is: a 3-day prohibition of all movements of susceptible animals after first detection (standstill), 3 km protection zones and 10 km surveillance zones around every detected farm, and culling of all animals in detected farms and their high risk contacts.

The results of the model are not meant to be interpreted as a strictly quantitative representation of reality. The application of models to decision-making, in general, should serve primarily as a means for comparing the effectiveness of different control measures, and assessing the comparative magnitude of various scenarios to understand the main outbreak drivers and the most important control targets (16). While we do not expect the model to tell us the exact number of herds that would be affected by a FMD outbreak in Sweden, for instance, the range of results evaluated gave us the expected dimension of the problem, in particular when compared among scenarios within this work, and also when compared to results from other countries.

Our results are a direct contrast to those observed when the same model was applied in Denmark, where the adoption of additional measures such as protective vaccination and ring depopulation were concluded to be cost-effective on most scenarios of spread (12). The contrasting conclusions, however, increase confidence that the results observed are not an artifact of the model, and highlight the impact that the specific characteristics of the Swedish livestock structure had in the model. In comparison to Denmark, Sweden is characterized by a low density of farms, with much smaller herd sizes on average, and most particularly, a small pig industry (23–25). Many farms also have very limited trade of live animals (26). In Finland, where cattle and pig farms are also typically family owned and small in size compared to the rest of Europe, and where the livestock industry has also been decreasing in recent years, results of a risk assessment published in 2011 were similar to the ones presented here (27). The authors concluded that a possible FMD outbreak in Finland would be controlled within 5 weeks of introduction, affecting on average four farms, and even the larger expected outbreaks would involve few farms and be promptly controlled.

Another difference to the original Danish model is that a reduction in the number of indirect contacts between farms after outbreak detection was assumed. The assumption that people would reduce all unnecessary traffic from and to their farms, once an outbreak is known to be occurring in the country, is based on feedback from farmers (28). It is also informed by the experience of our group through several outbreaks (of diseases other than FMD) and in particular a change in behavior noted in the country during the FMD outbreak in the UK in 2001.

The model was scrutinized by individual evaluation of multiple iterations per scenario, and mapping of every modeled transmission event, including the mode of transmission (direct or indirect contact). This confirmed that epidemic size was mainly driven by infected cattle farms. It also confirmed the expected effect of long distance movements in keeping North and South of Sweden highly inter-connected (29).

The choice of a predefined detection date (21 days after seeding the infection) was based on extensive review of information from previous outbreaks performed by the Danish team that developed the DTU-DADS model (12, 13). Complementary work (not presented in this paper) trying to estimate the detection date based on the probability of animals showing clinical signs, and the documented efficacy of passive surveillance in Sweden, suggested that 21 days is a conservative assumption. Relaxation of this assumption, i.e., assuming a later detection, was the single parameter with the most impact in the epidemic size. Sensitivity analysis showed that each week of later detection generally doubled the expected total number of infected herds by the time the epidemic is controlled. The epidemic duration (i.e., from day of detection to day of detection of last infected herd), however, showed remarkable robustness when compared to the number of infected herds, and the median epidemic duration was increased by only 3–4 days when detection was delayed by 1 week, and another 4–5 days for an extra week of delay. This highlights that surveillance resources were rarely exceeded, and the base control measures modeled were sufficient to cope with outbreaks of dimensions much larger than what was considered the “typical outbreak scenario” for Sweden.

The DTU-DADS model (in the version used to carry out this work, 0.15) did not allow adjustment of the surveillance capacity along the outbreak, that is, surveillance resources are fixed for the whole period of the epidemic. The surveillance capacity used in this model was based on what the Swedish Board of Agriculture considered feasible to gather in the first 1–3 days after detection of the first suspicion (and therefore arguably at the same time or shortly after confirmation). In reality, the number of surveillance resources could be increased after a few days of outbreak control. In the “typical outbreak scenario,” only in exceptional epidemics the number of herds that needed to be visited for clinical surveillance was greater than the number of teams available for field visits (see Figure S2-3 in Supplementary Material), and a herd queue was generated. The culling capacity, however, was never exceeded. Most of the farms in the model had herds smaller than the daily culling capacity per team declared by the Swedish Board of Agriculture and used in the model. Moreover, the number of herds that needed to be culled in the same day was very small.

The relatively small expected outbreak size and low demand for surveillance resources in all scenarios resulted in a high observed efficacy of the base control measures. In all evaluated scenarios, even the most chaotic ones, an FMD epidemic is expected to be controlled within 3 weeks from the detection of the first case. The number of herds infected is small, and most of the surveillance effort needed will be to visit farms that fall into the surveillance zones around each infected farm, to rule out infection. Surveillance capacity was not often exceeded. In epidemics that took longer than 2 weeks to control, herds could eventually wait longer than 2 days to be visited by a surveillance queue. However, herds in queue were those that needed to be visited because they fell within the surveillance zone. Suspected farms and high risk contacts are given priority in the surveillance visiting list, and therefore can be visited on the day of detection/tracing, as long as the number of infected herds and their direct contacts is below the number of surveillance teams, as was the case in all scenarios evaluated.

The base control measures were not only predicted to be effective, they were also robust. Reductions of up to 40% in the efficacy of a single measure can be compensated if everything else is assumed to be working properly. The number of infected herds was more sensitive to failures in control than the expected epidemic duration, due to the reasons discussed above.

Direct contact and local spread were the main modes of disease transmission. The central role of direct contact transmission is expected (30). In this model, the high percentage of local spread transmission is a consequence of the way the model was set up. Individual herds are modeled independently, and transmission between herds in the same farm is enforced by setting a high probability of local transmission within 100 m. The model was obviously sensitive to the set probability of transmission for other distance radius. In this model transmission events are modeled individually, and the addition of a local spread component was meant only to reflect any residual transmission not accounted for after modeling direct and indirect contacts explicitly.

The worst-case scenarios observed were those related to multiple introductions at the same time, and delayed detection of introduction. Even in those cases, a reduction in the expected number of infected herds as a result of the application of vaccination could not be demonstrated. Preemptive depopulation had an effect in reducing the median number of infected herds when very large epidemics were modeled (multiple introductions and late detection). Considering, however, that this measure would double the median number of herds and total animals to be culled, cost–benefit analysis will be needed to determine whether the benefits of applying this measure would justify the costs both in resources and animal welfare. As the current results indicate that the effect of preemptive depopulation can only become relevant for very large epidemics, this measure should only be considered after a large number of infected herds have been detected. Models exploring scenarios of FMD spread in the UK and Denmark have shown that ring culling can have a positive effect in specific circumstances (17, 31). We have, as those authors, concluded that the effect is not very pronounced, and more extensive analysis will be needed to determine the exact conditions under which an outbreak may have become large enough to justify preemptive depopulation.

While the base control strategy recommended based on this work is expected to be effective, it should be highlighted that the overall costs to the society and governmental agencies, as well as the workload, should not be overlooked. Effective control is associated with prompt implementation of a contingency strategy that would require deployment of 40 field surveillance teams per day and capacity to destroy thousands of animals per day. And for the control to be efficient, additional teams on central and regional level are needed working with contact tracing, data analysis, dissemination of information, logistics, etc., although these functions have not been included as a limiting factor in the model.

In summary, a potential FMD outbreak in Sweden is expected to be small and controlled fast through a 3-day national standstill, application of surveillance zones around infected farms, and culling of all animals in detected farms and their high risk contacts. This result is based on the assumption that detection would not be delayed by more than 4–5 weeks after introduction, that these measures would be enforced quickly after detection, and that the effectiveness of these control measures can be expected to fall within the range of values evaluated in this work.

## Author Contributions

FD adapted the infectious disease model to Sweden, parameterized and ran the model, summarized results, and wrote the manuscript. MN, JF, and KS helped parameterize the model, set relevant scenarios to be evaluated, and analyze and interpret results. SW helped adapt the model to Sweden, prepare Swedish data, and evaluate model behavior. AB and TH wrote the initial infectious disease model, trained the group into using it, and helped inspect model behavior and interpret results once the model was adapted to Sweden.

## Conflict of Interest Statement

The authors declare that the research was conducted in the absence of any commercial or financial relationships that could be construed as a potential conflict of interest.
