# Supplementary material for: Evaluation of Strategies to Control a Potential Outbreak of Foot-and-Mouth Disease in Sweden
Source: Front Vet Sci. 2017 Jul 24;4:118. doi: 10.3389/fvets.2017.00118 (PMC5523145; doi:10.3389/fvets.2017.00118)
Supplement: Supplementary file 1 [file presentation_1.pdf]

*Supplementary Material 1 – further details on METHODS*

# Evaluation of strategies to control a potential outbreak of Foot-and-Mouth disease in Sweden

**Fernanda C. Dórea*[1], Maria Nöremark[1], Stefan Widgren[1], Jenny Frössling[1], Anette Boklund[2], Tariq Halasa[2], Karl Ståhl[1]**

*** Correspondence:** Corresponding Author: fernanda.dorea@sva.se

## 1 Model parameters (inputs)

### 1.1 Index herd – where disease spread starts

FMD is introduced in the model by infecting one initial herd in the country, according to the disease spread scenario chosen (further details below). We have also modified the original Danish model to allow introduction in multiple herds at the same time.

### 1.2 FMD transmission parameters

All parameters referring to characteristics of the virus, infection and transmission were kept as in the Danish model, since those values were based on extensive literature research by that group (Boklund et al. 2013 and Halasa et al. 2014) and/or the group at UCDavis who developed the original model (Bates et al. 2003).

#### 1.2.1 Direct contact

When an infected animal is introduced in a new herd, the model draws from a random distribution to decide whether the virus is going to successfully spread into the animals in the new herd. The distribution is a pert distribution with a most likely value of 98%, but which can vary from 95% to 100%. The source of this value is not given in Boklund et al. 2013.

#### 1.2.2 Indirect contacts

Indirect contacts refer to the movement of people between farms. The model simulates two types of indirect contacts: medium risk contacts (professionals who come to the farm and have contact with animals) and low risk contacts (other visitors or trucks). Namely, medium risk contacts considered are: veterinarians, artificial inseminators and milk controllers. Low risk contacts are: non-professional visitors in general, rendering trucks, feed trucks, milk tanker routes and slaughter (trucks picking up animals in multiple farms in one single trip to the abattoir). Slaughter trucks are modelled individually, as shown in Table S1. All others are modelled generally as “medium risk contacts” and “low risk contacts”. A specific probability of visiting the farm daily was calculated for dairy tankers, but the probability of transmission associated with this activity is the same as used for low risk contacts.

TableS 1 lists the probability of transmission associated with each of these visitor types, that is, the probability that if one of these contacts visited an infected farm in the same day, they will successfully introduce the FMD virus into a susceptible farm being visited.

**TableS 1.** Probability of FMD virus transmission associated with different indirect contact types, based on the Danish model**.**

| Indirect contact type | Probability of transmission |
|---|---|
| Slaughter truck carrying pigs | Before detection: Pert(0.005, 0.175, 0.35)<br>After detection: Pert (0.002, 0.1, 0.06) |
| Slaughter truck carrying ruminants | Before detection: Pert (0.1, 0.5, 0.9)<br>After detection: Pert (0.002, 0.06, 0.1) |
| Low risk contact | Pert (0.005, 0.175, 0.35) |
| Medium risk contact | RUMINANTS: Pert (0.1, 0.5, 0.9)<br>PIGS (Hobby): Pert (0.1, 0.35, 0.9)<br>PIGS (others) : Pert (0.05, 0.2, 0.9) |

### 1.2.3 Local spread

The information below was transcribed from the supplementary material of Boklund et al. 2013 (Danish model):

> *Local spread is defined as cases within a certain radius which cannot be explained by other spread types. In descriptions of an epidemic, the following could be included in "local spread": spread by flies, birds, mice and rats, short distance windborne spread, neighbour visits (not registered) and animal movements which were not registered. In the simulation model, we know all movements (persons and animals) and therefore only windborne spread and birds etc. are considered as local spread. We simulated local spread within 3 km of herds with the distribution described in table [below]. The table should be read as there was a probability of disease spread of 0.95 from 0-100 meter, 0.012 from 100 meter to 1 km around the infected farm on etc.*

| *Distance (km)* | | | |
|---|---|---|---|
| *0.1* | *1* | *2* | *3* |
| *0.95* | *0.012* | *0.004* | *0.001* |

A particular issue with the model used by Boklund et al., and adopted in this work, is that animal herds are modelled individually. That is, if a single farm or owner has multiple herds, for instance a pig and a cattle herd, then each of those herds are modelled individually in the model. Besides the

estimated probabilities for 1, 2 and 3 km, the probability of transmission created for a 100m distance was deliberately inserted to account for transmission between herds in the same property.

.

### 1.2.4 Infectivity

If an infected animal is introduced into a new herd, the probability that the virus is transmitted to the new herd was discussed above (sections 1.2.1 and 1.2.2). If spread is successful, the next step for the model is to decide how many animals get infected per day, which is important to determine: when clinical signs will be noticed (allowing detection) and what is the probability of spreading the virus further from this newly infected herd, by direct or indirect contacts.

The information below was transcribed from the supplementary material of Boklund et al. 2013 (Danish model).

> *Animals in an infected herd will go through a latent period, a subclinical period and a clinical period. The animals are infectious during the subclinical and the clinical period. However, non-milking cattle herds and sheep herds was assumed to be infectious until day 21 (included), because of their limited herd size. When a herd gets infected, a within-herd spread will occur. In the DTU-DADS model, the latent period was modelled using a Poisson distribution with a lambda of 3.59, 3.07 and 4.79 for cattle, swine and sheep, respectively. The subclinical period was also modelled using a Poisson distribution with lambda of 2.04, 2.27 and 2.16 for cattle, swine and sheep, respectively (Mardones et al., 2010). This within-herd module will, for each herd, determine the proportion of infected animals.*
>
> *The distributions used to model time from infection to clinical signs (incubation period) was based on a meta-analysis of infection stages by Mardones et al. (2010). The articles included in the meta-analysis by Mardones et al. (2010) were all transmission experiments. In an outbreak situation, herds will be infected through different sources, some from import of animals already infected, some from transfer of virus that is taken up from one or several animals. Based on direct contacts, cattle would normally show clinical signs within 2-4 days (pers.communication Graham Belsham) and pigs within 72 hours (Alexandersen et al., 2003). As infected herds in the models are infected through either direct or indirect contacts, we combined the information.*

TableS 2 shows the final distribution of time from infection to clinical signs used in the Danish model.

**TableS 2.** Cumulative probability of an animal showing clinical signs, each day after infection (incubation period), in the infectious disease model described in Boklund et al. 2013.

| Species | Day | | | | | | | | | | | | |
|---|---|---|---|---|---|---|---|---|---|---|---|---|---|
| | 1 | 2 | 3 | 4 | 5 | 6 | 7 | 8 | 9 | 10 | 11 | 12 | 13 |
| Cattle | 0.052 | 0.153 | 0.31 | 0.495 | 0.669 | 0.805 | 0.897 | 0.951 | 0.979 | 0.992 | 0.998 | 1 | |
| Pigs | 0.108 | 0.27 | 0.475 | 0.669 | 0.816 | 0.909 | 0.96 | 0.984 | 0.994 | 0.998 | 0.999 | 1 | |
| Sheep, | 0.011 | 0.041 | 0.108 | 0.217 | 0.36 | 0.517 | 0.664 | 0.786 | 0.876 | 0.935 | 0.97 | 0.989 | 1 |

goat

### 1.2.5 Time to detection if clinical signs are present

The information below was transcribed from the supplementary material of Boklund et al. 2013 (Danish model).

> *The number of days before the herd would be detected is sampled from a distribution with the following probabilities for cattle and swine 0.085, 0.17, 0.51, 0.19, 0.06 and 0.07 for days from 1 to 6, respectively. For sheep herds the probabilities for days from 1 to 9 were 0.02, 0.04, 0.27, 0.29, 0.14, 0.12, 0.08, 0.05, and 0.12 respectively. Herds could however be detected faster though zone surveillance or tracing.*

TableS 3 gives the resulting cumulative probabilities of detection per day, as well as the adjusted probabilities in surveillance zones, as reported for the Danish model.

**TableS 3.** Probabilities of detecting herds showing clinical signs of FMD, after detection of the first herd (transcribed from Boklund et al., 2013, supplementary material).

| Days from clinical signs | Probability | | | |
|---|---|---|---|---|
| | Cattle & pigs | Sheep/goat | Cattle & pigs in zones or traced | Sheep/goat in zones or traced |
| -4 | 0 | 0 | 0 | 0.5 |
| -3 | 0 | 0 | 0 | 0.5 |
| -2 | 0 | 0 | 0 | 0.5 |
| -1 | 0 | 0 | 0 | 0.5 |
| 0 | 0 | 0 | 0 | 0.5 |
| 1 | 0.087 | 0.018 | 0.175 | 0.5 |
| 2 | 0.175 | 0.035 | 0.699 | 0.5 |
| 3 | 0.699 | 0.308 | 0.873 | 0.5 |
| 4 | 0.873 | 0.600 | 0.932 | 1 |
| 5 | 0.932 | 0.738 | 1 | 1 |
| 6 | 1 | 0.860 | 1 | 1 |
| 7 | 1 | 0.939 | 1 | 1 |
| 8 | 1 | 0.988 | 1 | 1 |
| 9 | 1 | 1 | 1 | 1 |

## 1.3 Parameters aimed at representing the Swedish animal population

To model FMD spread, the model requires that every herd in the country be listed individually, with geographical coordinates given. The way the model deals with farms containing multiple animal species is to list those herds individually – for instance a farm containing cattle and pigs would be listed twice, once for their cattle herd, and once for their pig herd. However since the coordinates of the two herds would be the same, the chance of spread from one herd to the other would be 95% (chance of local spread within 100m, see section 1.2.3).

For the Swedish model, we used herd information from the national registry database that the SJV makes available to SVA. We used the most recent information given to us by the SJV. We also used movement records from 2013 to calculate movement probability, distance, and to estimate the size of herds, as detailed below.

### 1.3.1 Cattle herds

Stefan Widgren (SVA) has been working in a spread model for VTEC (Verotoxin-producing *Escherichia coli*), using information from cattle herds. He had used the information available from the Swedish Board of Agriculture (SJV) regarding all the cattle herds in Sweden, crossing that information with the national registry of animal movement in order to calculate the size of each herd in 2013. For herds with missing geographical coordinates, Widgren assigned a random coordinated within the area referring to the farm's postal code.

We imported the information available in the VTEC project, and used the herd size estimated at the first day of 2013. Farms with a herd size of zero were excluded from the model. A total of 23 247 cattle herds were kept in the model, but most of them were small herds, as shown in FigureS 1. Only 220 herds had more than 500 cattle, with a maximum herd size of 2783. FigureS 1 also shows the distribution of the herds in Sweden.

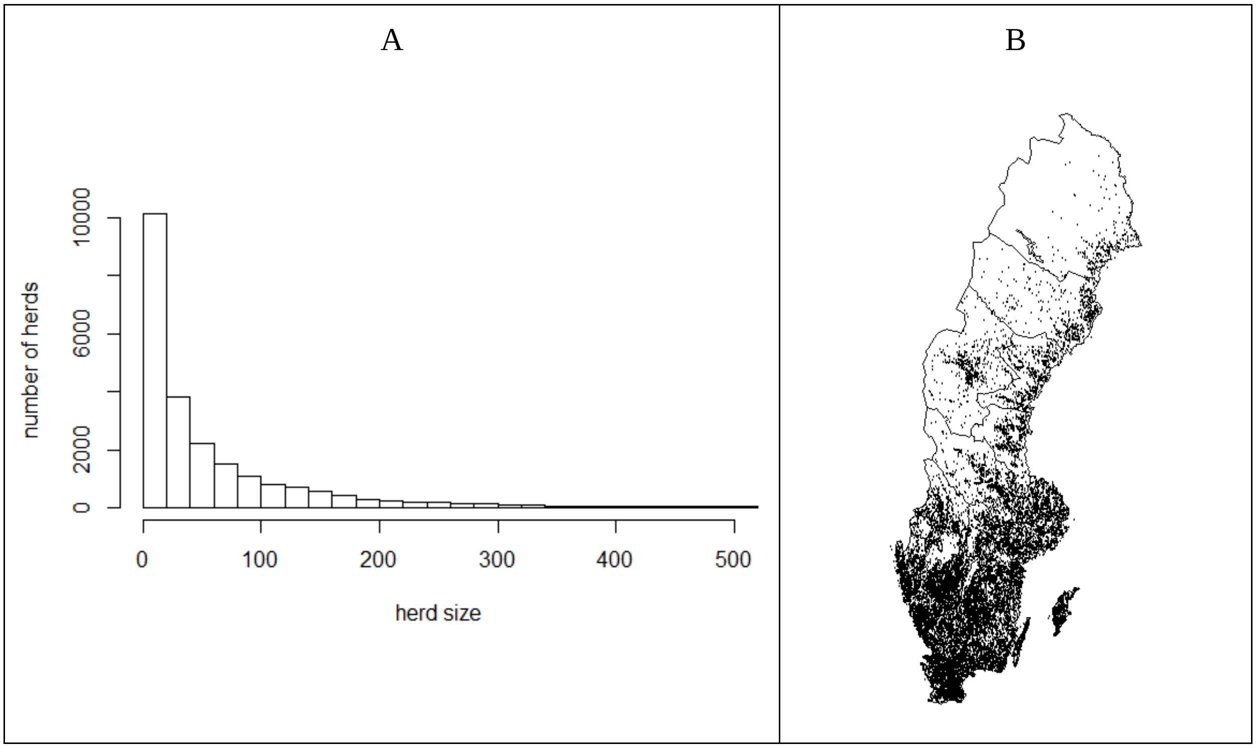


**FigureS 1.** Cattle herds used in the Swedish FMD spread model. A: herd size distribution, censored at 500 (actual maximum size equal to 2783). B: geographical coordinates of all 23 247 herds in the model.

Because of the indirect contact structure the model required herds to be classified into "milking" and "non-milking". This is mainly to determine whether the herd should be visited by a milk tank truck. A list of all "milking herds" was acquired through a Salmonella investigation project by Estelle Ågren (SVA). A total of 3 427 herds were listed as "milking".

### 1.3.2 Swine herds

Information about swine herds was taken from the available data provided by the SJV to SVA. Herds were listed as “Active”, “Incomplete” or “Inactive”. We kept all herds with status “Active” or “Incomplete”. Herds with missing geographical coordinates were assigned a random location within their declared postal code.

No precise information was available regarding the herd size. Therefore we used their declared “maximum capacity” as the herd size. For herds with herd size equal to zero, or missing information, we referred to the movement database in 2013. Herds with no size information, and which had no declared movement in 2013 were excluded from the model. The final number of herds in the model was 955. Figure S2 shows the distribution of herd sizes (the small insert focuses on the herds up to 1000 animals), and their geographical distribution.

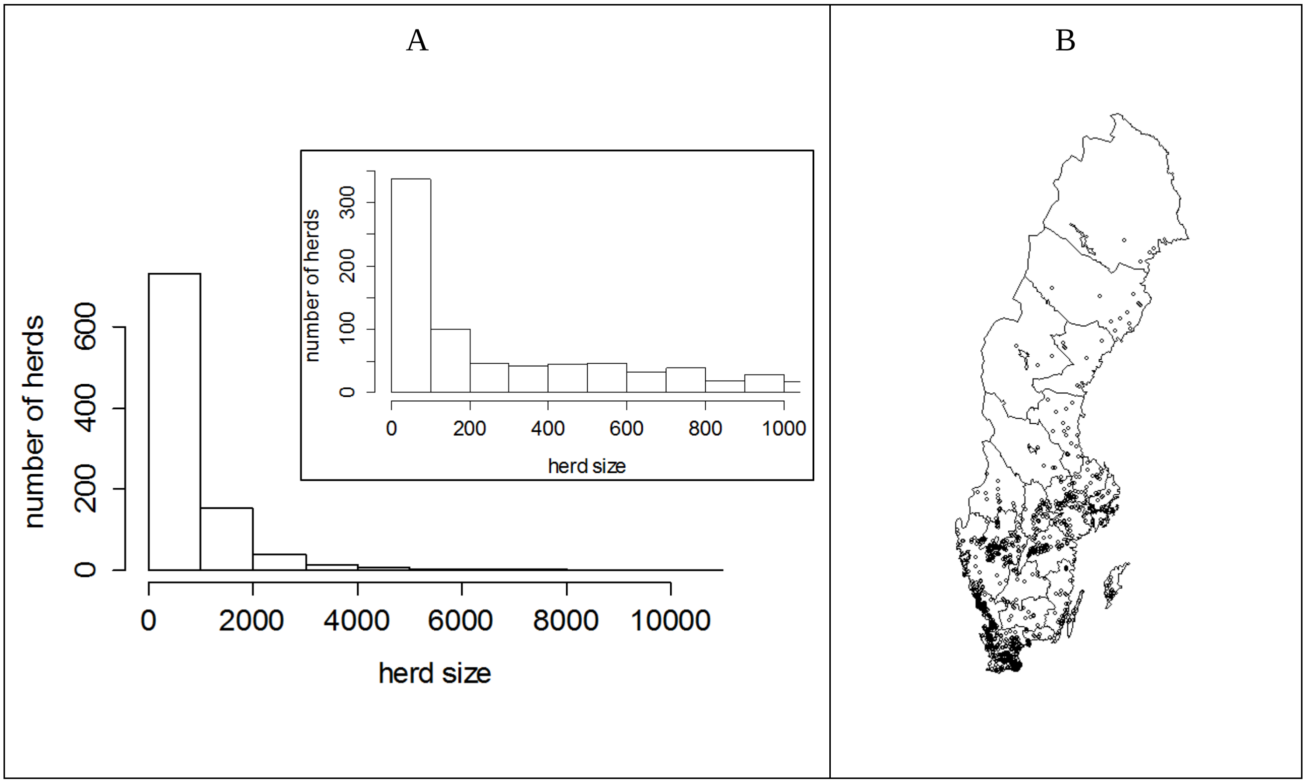


**Figure S2.** Swine herds used in the Swedish FMD spread model. A: herd size distribution, with the insert on top focusing on the herds up to 1000. B: geographical coordinates of all 955 herds in the model.

To classify swine herds into different types, we also used the information available at the national registry from SJV available at SVA. We were not successful in getting more detailed information regarding individual herd types from other sources of data, except for a list of KRAV herds from Jord På Trynet. The classification into herd types only affects the model in what regards the contact structure: we calculate a probability that animals and visitors would go from one type of herd, to any other type of herd. No other differences were programmed into the model due to herd type, since the probability of transporting animals and sending animals to slaughter, for instance, is calculated individually for every single herd, and doesn’t depend on herd type. Herds classified as “other” were

assumed to be equivalent to the risk category of “hobby” in the original Danish model, as detailed information about these herds’ production practices were not available.

Table S4 shows the herd types used and the number of herds classified into each of these types. Not all herds were used as seeding herds for epidemics, but their individual characteristics affect disease spread in the model once an epidemic is ongoing.

**Table S4.** Swine herd types in the Swedish model.

| Original code in the SJV database | Herd type | Number of herds |
|---|---|---|
| -- * | KRAV (integrated) | 11 |
| -- * | KRAV (not integrated) | 6 |
| AVEL | Multiplying herd | 29 |
| GYLT | Multiplying herd | 16 |
| SLAK | Fattening | 257 |
| SMA | Weaners | 255 |
| OVR | Others | 111 |
| INTE | Integrated | 180 |
| SUNA | Sow hers | 15 |
| SUSA | Sow herds | 75 |

*Information about KRAV farms came from Jord På Trynet.

### 1.3.3 Sheep and goats

Sheep and goat herds are treated as the same in the model. We got information regarding these herds from the national registry made available to SVA by the SJV. For sheep and goats we considered that it was likely not to have animal movement for a year, so we did not exclude herds without declared movement in 2013. We included all herds listed as “Active” or “Incomplete”. A total of 14 885 herds were included in the model, mostly small herds as shown in Figure S3. The maximum herd size was 1 658. Figure S3 also shows the geographical distribution of the herds, but please note that, as for cattle and pigs, the geographical coordinates for herds with missing information were randomly generated based on their postal code.

| A | B |
|---|---|

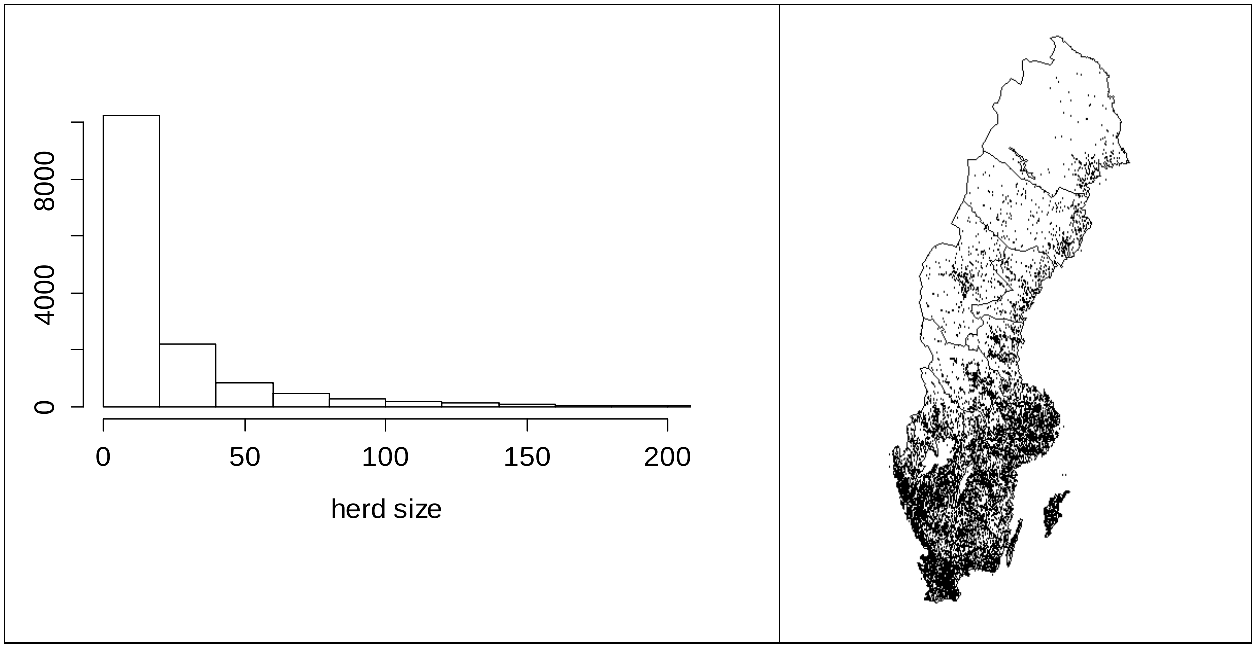


**Figure S3.** Sheep and goats herds used in the Swedish FMD spread model. A: herd size distribution, censored at a maximum 200 animals (the actual maximum herd size was 1658). B: geographical coordinates of all 14 885 herds in the model**.**

In the Danish model, sheep and goat herds were classified into “hobby” and “non-hobby” based on their size – herds with 40 animals or less were classified as hobby. Nöremark, Frössling and Lewerin (2010) used the 85% percentile of the distribution of herd sizes to decide which herds were hobby. The 85% percentile in our data was 43, which corroborates the cut-off number used in the Danish model, which was therefore kept. Based on this cut-off, 12628herds were classified as hobby, and 2257 as non-hobby. This classification only affects the model in determining the chance that animals and people leaving a herd of one of those types, will go to a herd of another type. But the probability of transporting animals is calculated individually for every single herd in Sweden, and does not depend on herd type.

### 1.3.4 Probability of moving animals

The probability of animals being moved out of a herd, in any given day, was calculated individually for every single herd in Sweden. This probability was used as the mean of a Poisson distribution that is used to decide, every day during the simulation, whether each individual herd will trade animals to another farm or send animals to slaughter.

We used the national movement registry (CDB database) to calculate this mean as described in the Danish model: for each individual herd a mean was calculated by dividing the number of days with any animal movement in 2013 by 365. That is, for each individual herd, we extracted all animal movements declared in 2013 for which that herd was the herd of origin. We then removed duplicated days.

A mean for animal movements to other farms, and a mean for animal movements to slaughter was calculated for every herd in the model. The distributions found in the data are detailed in Table S5.

**Table S5.** Distribution of average number of daily movements for each herd in the model, grouped by species.

| Species | 5% | 25% | 50% | 75% | 95% | Maximum |
|---|---|---|---|---|---|---|
| Cattle | 0.0000 | 0.0000 | 0.0027 | 0.0082 | 0.0301 | 0.3699 |
| Swine | 0.0027 | 0.0082 | 0.0383 | 0.0818 | 0.1871 | 0.4603 |
| Sheep/goats | 0.0000 | 0.0000 | 0.0000 | 0.0028 | 0.0109 | 0.0931 |

To evaluate the effect of starting the epidemic in herds with different probabilities of trading animals, we picked index herds based on their daily trading probability, as detailed in Table S6.

**Table S6.** Cut-offs used to classify herds based on their daily average of animal movement.

| Species | Low trade herds | Medium trade herds | High trade herds |
|---|---|---|---|
| Cattle | <0.001 | 0.002 – 0.003 | >0.04 |
| Swine | <0.003 | 0.03-0.05 | >0.18 |
| Sheep/goats | -- | -- | >0.01 |

### 1.3.5 Region

The main expected difference between regions in Sweden, which could affect the patterns of FMD dissemination, were the distances between farms. To account for those differences, we have defined two regions: North and South. As a reference, we used the Nomenclature of Units for Territorial Statistics (NUTS), which defines 8 national areas for Sweden, and 3 regions (NUTS1), as shown in Figure S4. NUTS2 and NUTS1 classifications for Sweden. The south-most (Götaland) and middle (Svealand) regions shown in Figure S4 were defined as “South” region in the model. Norrland was used to represent “North”.

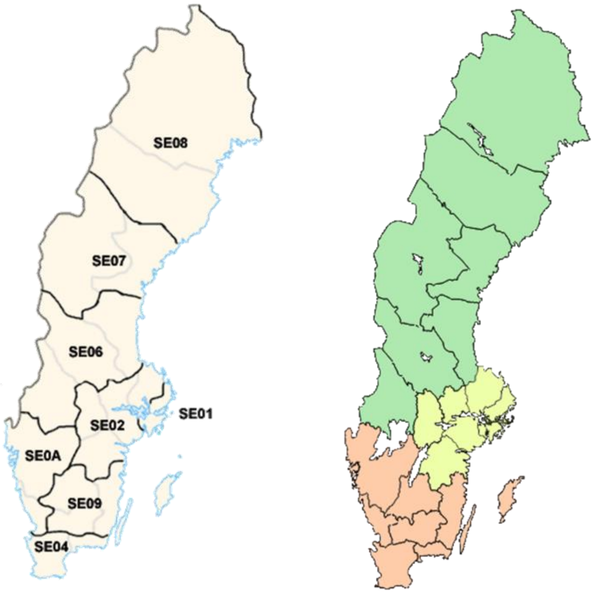


**Figure S4.** NUTS2 and NUTS1 classifications for Sweden. In the Swedish FMD spread model the south most (Götaland) and middle (Svealand) regions were classified as “South”.

### 1.3.6 Distance of movement

In the simulation model, every day it is decided, based on probability distributions, whether each individual herd will send animals to other farms or to slaughter, as described in section 1.3.4. Once the model “decides” that a farm will move animals, the distance those animals are going to be moved (what is the distance of the origin to the destination farm or slaughterhouse) must be determined. The Danish model requires as input that probabilities are given for a number of distance bands – that is, what is the probability that the destination farm will be in a 10km radius, or 20 km radius, and so on.

We used the CDB database to calculate the Euclidian distance between origin and destination farm for every animal movement event declared in 2013, and calculate such probabilities. These probabilities were calculated separately for animal trade and slaughter, for each type of animal (cattle, pigs and sheep) and for each region (north and south).

Table S7 lists the probability associated with each distance band, for every type of animal movement considered in the model, for regions North and South.

**Table S7.** Probability of moving animals in each distance band, for all different types of animal movements considered in the model, and for regions North and South.

| | Probability of movement in each distance band | | | | | | | | | | | |
|---|---|---|---|---|---|---|---|---|---|---|---|---|
| | Cattle | | | | Pigs | | | | Sheep | | | |
| Distance (km) | Trade | | Slaughter | | Trade | | Slaughter | | Trade | | Slaughter | |
| | South | North | South | North | South | North | South | North | South | North | South | North |

| | | | | | | | | | | | | |
|---|---|---|---|---|---|---|---|---|---|---|---|---|
| 0-1 | 0.0418 | 0.0516 | 0.0006 | 0.0000 | 0.0014 | 0.0036 | 0.0117 | 0.1029 | 0.0223 | 0.0258 | 0.0093 | 0.0148 |
| >1-3 | 0.1071 | 0.0776 | 0.0013 | 0.0008 | 0.0084 | 0.0170 | 0.0085 | 0.0290 | 0.0427 | 0.0413 | 0.0082 | 0.0154 |
| >3-10 | 0.2582 | 0.2071 | 0.0160 | 0.0087 | 0.0678 | 0.0386 | 0.0610 | 0.0351 | 0.1089 | 0.1263 | 0.1035 | 0.0444 |
| >10-15 | 0.0907 | 0.1050 | 0.0222 | 0.0065 | 0.0438 | 0.0081 | 0.0383 | 0.0080 | 0.0615 | 0.0689 | 0.0844 | 0.0512 |
| >15-20 | 0.0688 | 0.0678 | 0.0269 | 0.0117 | 0.0300 | 0.0063 | 0.0432 | 0.0413 | 0.0560 | 0.0545 | 0.0952 | 0.0481 |
| >20-30 | 0.0886 | 0.0866 | 0.0548 | 0.0213 | 0.0644 | 0.0798 | 0.0852 | 0.0037 | 0.0825 | 0.1269 | 0.1502 | 0.1276 |
| >30-40 | 0.0630 | 0.0599 | 0.0557 | 0.0257 | 0.0785 | 0.0466 | 0.0612 | 0.0813 | 0.0650 | 0.0568 | 0.1444 | 0.1245 |
| >40-50 | 0.0477 | 0.0343 | 0.0500 | 0.0253 | 0.0853 | 0.0260 | 0.0656 | 0.0407 | 0.0560 | 0.0643 | 0.1018 | 0.0931 |
| >50-60 | 0.0366 | 0.0337 | 0.0428 | 0.0214 | 0.0532 | 0.0215 | 0.0785 | 0.0345 | 0.0465 | 0.0482 | 0.0683 | 0.0641 |
| >60-70 | 0.0294 | 0.0235 | 0.0458 | 0.0284 | 0.0757 | 0.0072 | 0.0324 | 0.1571 | 0.0331 | 0.0299 | 0.0502 | 0.0364 |
| >70-80 | 0.0238 | 0.0210 | 0.0423 | 0.0303 | 0.0424 | 0.0502 | 0.0727 | 0.0863 | 0.0461 | 0.0281 | 0.0303 | 0.0382 |
| >80-90 | 0.0191 | 0.0119 | 0.0430 | 0.0179 | 0.0373 | 0.0825 | 0.0632 | 0.0715 | 0.0371 | 0.0166 | 0.0279 | 0.0339 |
| >90-100 | 0.0170 | 0.0117 | 0.0374 | 0.0266 | 0.0194 | 0.0484 | 0.0333 | 0.0604 | 0.0300 | 0.0161 | 0.0166 | 0.0321 |
| >100-110 | 0.0123 | 0.0118 | 0.0323 | 0.0226 | 0.0168 | 0.0628 | 0.0191 | 0.0197 | 0.0311 | 0.0247 | 0.0175 | 0.0364 |
| >110-120 | 0.0111 | 0.0125 | 0.0365 | 0.0405 | 0.0195 | 0.1139 | 0.0220 | 0.0086 | 0.0296 | 0.0201 | 0.0144 | 0.0382 |
| >120-130 | 0.0085 | 0.0094 | 0.0355 | 0.0332 | 0.0202 | 0.0735 | 0.0375 | 0.0191 | 0.0317 | 0.0224 | 0.0150 | 0.0160 |
| >130-140 | 0.0077 | 0.0075 | 0.0352 | 0.0228 | 0.0400 | 0.0556 | 0.0145 | 0.0394 | 0.0197 | 0.0115 | 0.0118 | 0.0197 |
| >140-150 | 0.0060 | 0.0057 | 0.0338 | 0.0213 | 0.0280 | 0.0072 | 0.0351 | 0.0185 | 0.0184 | 0.0080 | 0.0089 | 0.0203 |
| >150-160 | 0.0058 | 0.0068 | 0.0296 | 0.0225 | 0.0220 | 0.0422 | 0.0332 | 0.0105 | 0.0146 | 0.0212 | 0.0089 | 0.0253 |
| >160-170 | 0.0060 | 0.0082 | 0.0323 | 0.0231 | 0.0150 | 0.0099 | 0.0147 | 0.0006 | 0.0139 | 0.0264 | 0.0045 | 0.0080 |
| >170-180 | 0.0052 | 0.0060 | 0.0288 | 0.0282 | 0.0150 | 0.0152 | 0.0200 | 0.0228 | 0.0100 | 0.0109 | 0.0032 | 0.0099 |
| >180-190 | 0.0045 | 0.0066 | 0.0245 | 0.0221 | 0.0130 | 0.0305 | 0.0038 | 0.0074 | 0.0106 | 0.0138 | 0.0087 | 0.0228 |
| >190-200 | 0.0034 | 0.0063 | 0.0227 | 0.0176 | 0.0092 | 0.0018 | 0.0070 | 0.0037 | 0.0078 | 0.0276 | 0.0038 | 0.0259 |

| | | | | | | | | | | | | |
|---|---|---|---|---|---|---|---|---|---|---|---|---|
| >200-210 | 0.0036 | 0.0043 | 0.0173 | 0.0125 | 0.0127 | 0.0009 | 0.0276 | 0.0524 | 0.0173 | 0.0172 | 0.0037 | 0.0148 |
| >210-220 | 0.0028 | 0.0038 | 0.0179 | 0.0186 | 0.0109 | 0.0682 | 0.0148 | 0.0074 | 0.0098 | 0.0195 | 0.0038 | 0.0099 |
| >220-230 | 0.0028 | 0.0041 | 0.0186 | 0.0107 | 0.0092 | 0.0009 | 0.0074 | 0.0000 | 0.0172 | 0.0063 | 0.0016 | 0.0179 |
| >230-240 | 0.0024 | 0.0023 | 0.0192 | 0.0032 | 0.0246 | 0.0009 | 0.0178 | 0.0000 | 0.0173 | 0.0103 | 0.0005 | 0.0037 |
| >240-250 | 0.0022 | 0.0022 | 0.0211 | 0.0149 | 0.0202 | 0.0018 | 0.0146 | 0.0080 | 0.0137 | 0.0086 | 0.0007 | 0.0012 |
| >250-300 | 0.0237 | 0.1107 | 0.1561 | 0.4618 | 0.1163 | 0.0789 | 0.0561 | 0.0302 | 0.0497 | 0.0476 | 0.0027 | 0.0062 |

### 1.3.7 Probability of sending animals to other herd types.

For every animal movement event simulated in the model, the model must also decide to what kind of herd the animals are going to be sent to. We again used the CDB data (animal movement registry) to calculate the probability of sending animals from each herd type to every other herd type, based on all movements registered in 2013. We calculated, for instance, how many of the cattle movements in 2013 for which the source herd was a milking herd, had as destination another milking herd, or a non-milking herd.

The resulting probabilities used in the model, for cattle swine and sheep, respectively, are listed in Tables Table S8, Table S9 and Table S10.

**Table S8.** Probability of any given cattle movement starting in a milking or non-milking herd, having as destination a herd of the same type of a different type.

| | **Destination** | |
|---|---|---|
| **Origin** | **Not-Milking** | **Milking** |
| **Milking** | 0.6337 | 0.3663 |
| **Not-Milking** | 0.8884 | 0.1116 |

**Table S9.** Probability of any given pig movement starting in a specific herd type and ending in every other herd type, in the North and South of Sweden.

| | Destination | | | | | | | | | |
|---|---|---|---|---|---|---|---|---|---|---|
| Origin | KRAV-integrated | KRAV-not integrated | multipliers (AVEL) | multipliers (GYLT) | Fattening | Weaners | Others | Integrated | Satellite (SUNA) | Satellite (SUSA) |
| KRAV- | 0.1077 | 0.0269 | 0.0000 | 0.0000 | 0.2269 | 0.0000 | 0.6385 | 0.0000 | 0.0000 | 0.0000 |

| | | | | | | | | | | |
|---|---|---|---|---|---|---|---|---|---|---|
| integrated | | | | | | | | | | |
| KRAV-not integrated | 0.0206 | 0.0000 | 0.0000 | 0.0000 | 0.0123 | 0.0000 | 0.9671 | 0.0000 | 0.0000 | 0.0000 |
| multipliers (AVEL) | 0.0014 | 0.0055 | 0.0565 | 0.0634 | 0.1612 | 0.1515 | 0.4091 | 0.0675 | 0.0193 | 0.0647 |
| multipliers (GYLT) | 0.0095 | 0.0061 | 0.0007 | 0.0075 | 0.1913 | 0.2873 | 0.1879 | 0.1920 | 0.0796 | 0.0381 |
| Fattening | 0.0000 | 0.0003 | 0.0037 | 0.0008 | 0.0702 | 0.0198 | 0.8552 | 0.0211 | 0.0200 | 0.0089 |
| Weaners | 0.0037 | 0.0000 | 0.0006 | 0.0000 | 0.5619 | 0.0156 | 0.3270 | 0.0614 | 0.0162 | 0.0136 |
| Others | 0.0000 | 0.0000 | 0.0206 | 0.0000 | 0.1719 | 0.0234 | 0.7015 | 0.0248 | 0.0468 | 0.0110 |
| Integrated | 0.0011 | 0.0024 | 0.0160 | 0.0106 | 0.2047 | 0.0434 | 0.6618 | 0.0311 | 0.0192 | 0.0097 |
| Satellite (SUNA) | 0.0000 | 0.0000 | 0.0000 | 0.0000 | 0.2173 | 0.0958 | 0.4004 | 0.0511 | 0.0000 | 0.2354 |
| Satellite (SUSA) | 0.0000 | 0.0000 | 0.0018 | 0.0000 | 0.4563 | 0.0269 | 0.2972 | 0.0359 | 0.0975 | 0.0843 |

**Table S10.** Probability of any given sheep/goat movement starting in a hobby or non-hobby herd, having as destination a herd of the same type of a different type, in the North and South of Sweden.

| | **Destination** | |
|---|---|---|
| **Origin** | **Not-Hobby** | **Hobby** |
| **Hobby** | 0.1640 | 0.8359 |
| **Not-Hobby** | 0.1691 | 0.8309 |

### 1.3.8 Indirect contacts

Besides the direct contacts characterized in sections 1.3.4 to 1.3.7 above, the model also simulates the indirect contact structure, that is, the movement of people between farms. We estimated the average number of medium and low risk contacts per day, for each species, based on data available from Nöremark et al., 2013. In the Danish model it was reported that only 25% of low risk contacts were associated with any risk of FMD transmission, based on the reference work of Nielen et al. (1996). We applied this correction t the number of calculated low risk visitors per day. The figures are listed in Table 9.

**Table 11.** Average number of visitors daily, to herds of each species and type.

| | Average number of visitors daily | | |
|---|---|---|---|
| | Medium risk contacts | Low risk contacts | Milk tank truck |

| | | | |
|---|---|---|---|
| Cattle milking | 0.1391 | 0.0905 | 0.1074 |
| Cattle not-milking | 0.0117 | 0.0521 | -- |
| Pigs | 0.0354 | 0.0822 | -- |
| Sheep | 0.0138 | 0.0684 | -- |

As listed above for direct contacts, in the case of indirect contacts the model must also decide whether a visitor leaving a farm will next go to a herd of the same type, or to a herd or another type. We were not able to find any studies that would allow estimation of these probabilities for the Swedish reality, so we kept the parameters used in the Danish model, as transcribed below form the supplementary material of Boklund et al. (2013):

*Medium risk contacts from cattle herds were modelled to most often have another cattle herd as the destination herd (88%), while we modelled 60% and 40% of the medium risk contacts to go to other herd types (cattle or sheep) from hobby and non-hobby pig herds, respectively. [...] From sheep herds, we assumed that 50% of the movements were to other sheep herds, while the other 50% were to pig or cattle herds.*

Please note that in the Swedish model the category "others" corresponds to the "hobby" category listed for the Danish model.

Lastly, for indirect contacts the model must also determine the distance traveled by each visitor. We were not able to have access to the data of any studies that investigated traveled distance by different types of visitors per day, therefore we kept the parameters used in the Danish model. Medium risk contacts were assumed to travel distances of 10, 20, 30 or 45km with probabilities of 20%, 20%, 20% and 40%, respectively. For low risk contacts the distance bands considered are up to 3km, 10, 20, 30 and 100km. The respective probabilities are 0.36, 0.29, 0.18, 0.05 and 0.12 in pig herds and 0.61, 0.3, 0, 0, and 0.9 for ruminants.

### 1.3.9 Number of farms in a slaughter truck route

We were unable to get precise estimates for Sweden, but Maria Nöremark (SVA) compared the usual number of animals transported to slaughter per event with the average size of transporting trucks, establishing that we could use the number from the Danish model (until better estimations are found). The following is transcribed from Boklund et al. (2013):

*The number of herds on a route differed between species. For swine, the number of herds visited by one vehicle ranged from 1 to 7, and 53% of all finisher transports was directly from one herd to the abattoir. [...] In the period after detection of the index herd, we assumed that transport of animals for slaughter would only be from one herd directly to the abattoir and with higher levels of bio-security. Therefore, after day 21, the number of direct contacts from this movement type was reduced to a Poisson distribution with*

*lambda=1.5 and the risk of transmission was reduced to 0.06 (Pert distribution between 0.002 and 0.1 with 0.06 as the most likely).*

For cattle farms, the Danish model uses a Poisson distribution with mean 5 as the expected number of farms included in each slaughter truck route.

## 1.4 Parameters related to epidemic control

All parameters listed above are mainly given as fixed input to the model. The parameters listed in this section are those that were changed from scenario to scenario, in order to evaluate their effect in the expected epidemic magnitude, and allow us to draw conclusions about the efficacy of different strategies. The entire range of values explored was listed in the main article (Table 1), and detailing these values makes more sense when we discuss the results of different control scenarios.

In this section we will just summarize any information needed to understand the meaning of these inputs, and how they are used in the model.

### 1.4.1 Epidemic detection

The model does not simulate which day the epidemic will be detected – rather, it requires as an input a decision regarding how many days, after introduction in the first herd, the epidemic will be detected. In the Danish model the base assumption is that detection will occur 21 days after introduction in the seed herd, referencing the epidemics in England in 2001 and in the Netherlands in 2001. We have used this as the base scenario, but also evaluated earlier and later detection.

### 1.4.2 Survey zones

Radial zones for control action are set around every infected farm that is detected. In any scenario, a 3km protection zone and a 10km surveillance zones are set around any detected farm for 30 days. All farms in this zone are prohibited from moving animals (the prohibition is considered to be 98% effective).

Every detected farm is depopulated the day after detection. As described in the Danish model (Boklund et al. (2013): "*These were first priority on the shared resource list, meaning that in case infected herds and neighbour herds or traced herds were waiting to be depopulated, infected herds would be depopulated first. Traced forward movements of animals to other herds [...] were depopulated. These were second priority on the shared resource list".* In the scenarios where radial depopulation was enforced (below), depopulation in zones was third in the priority list.

Then, every farm in the protection zone (3km around the infected farm) is put on a surveillance list. These farms are to be visited by surveillance teams depending on the capacity available – as many farms as possible are visited every day, and if the number of farms in the list is greater than the number of teams, they wait on the list until they can be visited. It is not possible, in the Danish model, to set varying resources along the epidemic. That is, it is not possible to start with a small number of teams and then increase. The number of surveillance teams must be fixed. But we have evaluated different scenarios, and discussed the use of resources.

Farms waiting to be visited are already considered closed, and the only spread that can occur is local spread. When a surveillance team is available to visit the farm, the model decides whether the team will detect clinical signs, should that farm be infected. This depends on whether the farm is infected, whether animals are already showing clinical sign, and the probability of detection when clinical

signs are present. All herds in the protection zone were assumed to be visited again before lifting the zone (so if clinical signs are not detectable in the first visit they could be detected on the second visit). The revisiting time was set to 14 days.

All farms in the surveillance zone (10km) are also put on the waiting list to be visited, but with secondary priority. The goal is to have farms in the protection zone visited 3-7 days after declaring the zones, and farms in the surveillance zone can wait 10-15 days.

If upon visit any farm is declared infected, the cycle starts again – the new farm is set to be depopulated the next day, and protection and surveillance zones are set around the newly detected farm. When zones set around 2 farms overlap, some farms which were already in the surveillance list will be assigned to the list again. In this case the farm does get visited a second time, but with lower priority in relation to farms who were not visited yet. The waiting time for the visit in this case was set to 7 days.

As mentioned before, farms can stay multiple days in the surveillance waiting list if there are not enough surveillance teams to visit all farms in the same day. Similarly, the model works based on a given culling capacity, and if extrapolated, farms wait in the culling queue.

#### 1.4.3 Animal Movement ban

Besides the movement restrictions applied in the survey zones for 30 days, a nationwide ban on all animal movements is enforced when the epidemic is first detected in the country, and considered to be 98% effective (Pert (0.95, 0.98,1)). The Danish model was not programmed to allow applying the ban separately on movement of live animals, and movement of animals to slaughter. The duration of this all-inclusive ban can however be changed (set to 3 days in the Danish model), and was explored in different scenarios.

#### 1.4.4 Tracing

When a farm is declared infected, the work of tracing all movement to and from the farm starts immediately. The model assumes it takes one day to trace all movements. All the farms which received animals <u>from</u> the infected farm are considered to be <u>high-risk contacts</u>. The standard behaviour of the model is to put those high-risk contacts in depopulation list immediately, but we also evaluated the effect of just putting them on the surveillance queue (so that a team can visit and decide if the farm is infected). All the farms which sent animals to the infected one, or which has established indirect contact with the infected farm, are put in the surveillance queue to be visited. The tracing of direct contacts was considered to be 98% effective, and the success of tracing indirect contacts was set to 80% for medium risk (professionals, such as veterinarians) and 50% for low risk.

#### 1.4.5 Changes in the patterns of indirect contacts

The Danish model established a ban on the movement of animals, but not on the movement of people. They did however consider that there would be a reduction in the movement of people between farms within the survey zones – that reduction was of about 80% for medium contact risks, and 30% for low risk contacts.

In Sweden, however, we did believe that people and farmers would change their behaviour once they know that FMD has been detected anywhere in the country – that is, these changes would not be

restricted to the survey zones. Therefore, we extended the changes applied to the survey zones to the whole country once the first herd is detected.

### 1.4.6 Ring depopulation

In the base model, depopulation is only applied to farms detected and to their high-risk contacts. When exploring additional control scenarios, it is also possible to apply culling to all farms within a radius from the infected farm.

It is also possible to set when this additional control measure will start to be applied – from the beginning of the epidemic (the next day after the first infected herd is detected in the country), only when the epidemic is not yet controlled after 14 days, or after the number of herds infected reaches a certain threshold.

### 1.4.7 Ring vaccination

Ring vaccination works in a way similar to ring depopulation – it is enforced in a radius around the infected farm, and it can be decided to start vaccination from the beginning of the epidemic, after a number of days, or when a certain number of infected herds is reached.

It is important to note that the Danish model was set up to use vaccination as a measure to stop spread while animals wait to be culled, that is, to "buy time" if we run out of resources to depopulate all herds immediately. Therefore, *all vaccinated herds are killed at the end of the epidemic, as the resources (surveillance teams) becomes available* (personal communication with Tariq Halasa).

All the parameters for setting up vaccination strategies were borrowed from the Danish model, and transcribed below:

*We used a weighted average for vaccine efficacy in cattle, based on an assumption that a herd would have 70% older and 30% younger animals. An efficacy of 0.71 (0.59–0.85) for calves (Halasa et al., 2011) and 0.3 for cows (Orsel et al., 2007) lead us to an estimate of 0.42 for cattle with a confidence interval of 0.39-0.47. For pigs and sheep we based our estimates on Halasa et al., leading us to an efficacy of 0.67 (0.51-0.87) and 0.59 (0.44-0.80) for pigs and sheep, respectively. […] the vaccine efficacy from a look-up table was used to model the immunity.*
***Table.*** *Time from vaccination to immunity after vaccination. The figures in the table will be multiplied on the probability of infection, when a source herd is exposing a vaccinated farm; therefore one equals no immunity and zero equals full immunity (ISP).*

| *Day after vaccination* | *1* | *2* | *3* | *4* | *5* | *6* | *7* | *8* | *≥9* |
|---|---|---|---|---|---|---|---|---|---|
| *Cattle* | *1* | *1* | *1* | *0.958* | *0.874* | *0.79* | *0.622* | *0.58* | |
| *Pigs* | *1* | *1* | *1* | *1* | *0.933* | *0.799* | *0.665* | *0.397* | *0.33* |
| *Sheep* | *1* | *1* | *1* | *0.941* | *0.823* | *0.705* | *0.469* | *0.41* | |

*Vaccinated herds would have a reduced probability of infecting other herds. Table [below] describes the probability distribution, over time, of how we assumed infectiousness to be reduced in vaccinated herds (personal communication, Anette Bøtner and Graham Belsham).*
***Table.*** *Infectivity after vaccination. The figures in the table are multiplied by the probability of infection when a vaccinated farm is exposing another farm; therefore one equals no immunity and zero equals full immunity (ISP only).*

| *Day after* | *1* | *2* | *3* | *4* | *5* | *6* | *7* |
|---|---|---|---|---|---|---|---|

| *vaccination* | | | | | | | |
|---|---|---|---|---|---|---|---|
| *Milking cows* | *0.013* | *0.067* | *0.193* | *0.27* | *0.359* | *0.443* | *0.1* |
| *Not milking* | *0.1* | *0.2* | *0.6* | *0.63* | *0.7* | *0.5* | *0.1* |
| *Pigs* | *0.002* | *0.067* | *0.116* | *0.167* | *0.238* | *0.263* | *0.314* |
| *Sheep Non-Hobby* | *0.014* | *0.014* | *0.071* | *0.167* | *0.19* | *0.179* | *0.044* |
| *Sheep - Hobby* | *0.05* | *0.05* | *0.25* | *0.495* | *0.455* | *0.375* | *0.095* |

| *Day after vaccination* | *8* | *≥9* | *10* | *11* | *12* | *13* | *14* | *>21* |
|---|---|---|---|---|---|---|---|---|
| *Milking cows* | *0.1* | *0.1* | *0.1* | *0.1* | *0.1* | *0.1* | *0.1* | *0.1* |
| *Not milking* | *0.1* | *0.1* | *0.1* | *0.1* | *0.1* | *0.1* | *0.1* | *0* |
| *Pigs* | *0.085* | *0.1* | *0.1* | *0.1* | *0.1* | *0.1* | *0.1* | *0.1* |
| *Sheep Non-Hobby* | *0.054* | *0.068* | *0.08* | *0.089* | *0.094* | *0.086* | *0.1* | *0* |
| *Sheep - Hobby* | *0.1* | *0.1* | *0.1* | *0.1* | *0.1* | *0.1* | *0.1* | *0* |

*In vaccination scenarios, all vaccinated herds were clinically surveyed at the time of vaccination and again between day 33 and 37 (most likely day 35) after creation of the vaccination zone […] The probability of detection was modelled to be the same as in surveillance within zones.*

## 2 References


Boklund, A.; Halasa, T; Christiansen, L.E.; Enøe, C. 2013. Comparing control strategies against foot-and-mouth disease: Will vaccination be cost-effective in Denmark? Preventive Veterinary Medicine 111: 206–219

Halasa, T.; Boklund, A.; Stockmarr, A.; Enøe, C.; Christiansen, L.E. 2014. A Comparison between Two Simulation Models for Spread of Foot-and-Mouth Disease. Plos One, 9:3.

Nielen, M., Jalvingh, A.W., Horst, H.S., Dijkhuizen, A.A., Maurice, H., Schut, B.H., Wuijckhuise, L.v., Jong, M.d., Van Wuijckhuise, L.A., De Jong, M.F., 1996. Quantification of contacts between Dutch farms to assess the potential risk of foot-and-mouth disease spread. Prev Vet Med 28, 143-158.

Nöremark, M; Frössling, J. and Sternberg Lewerin, S. 2013 A survey of visitors on Swedish livestock farms with reference to the spread of animal diseases. BMC Veterinary Research, 9:184
