# Supplementary material for: Evaluation of Strategies to Control a Potential Outbreak of Foot-and-Mouth Disease in Sweden
Source: Front Vet Sci. 2017 Jul 24;4:118. doi: 10.3389/fvets.2017.00118 (PMC5523145; doi:10.3389/fvets.2017.00118)
Supplement: Supplementary file 2 [file presentation_2.pdf]

Supplementary Material 2 – further details on RESULTS

Evaluation of strategies to control a potential outbreak of Foot-and-Mouth disease in Sweden

Fernanda C. Dórea*[1], Maria Nöremark[1], Stefan Widgren[1], Jenny Frössling[1], Anette Boklund[2], Tariq Halasa[2], Karl Ståhl[1]

*** Correspondence:** Corresponding Author: fernanda.dorea@sva.se

1 Additional tables

Table S2-1. Selected summary statistics for all the scenarios evaluated.

	Epidemic Duration			Number of Infected herds			Number of Visited herds			Number of culled herds			Number of culled Animals		
	Med	75%	95%	Med	75%	95%	Med	75%	95%	Med	75%	95%	Med	75%	95%
Cattle Milking South	11	15	22	8	12	20	381	572	909	8	12	20	460	846	1872
Cattle Not-Milk South	5	10	19	3	5	12	122	250	622	3	5	12	119	315	877
Pigs Sows South	9	13	21	6	9	15	261	417	803	6	9	16	1280	2736	7370
Pigs Fattening South	9	12	21	5	8	14	243	408	797	5	8	15	1718	3048	7393
Pigs Weaners South	9	13	22	6	9	16	300	484	876	6	9	16	927	2168	5594
Pigs multipliers South	10	14	21	7	11	18	342	543	915	7	11	18	987	2485	6884
Small Ruminants South	4	9	15	3	4	8	106	189	448	3	4	8	70	190	616
Cattle Milking North	10	14	22	7	11	19	195	351	734	7	11	19	373	613	1260
Cattle Not-Milk North	3	9	16	2	4	10	51	99	399	2	4	10	86	195	614
Pigs Sows North	8	12	18	5	7	12	164	280	511	5	7	12	1586	3002	6127
Pigs Fattening North	8	12	19	5	7	13	137	265	550	5	7	13	550	1610	4240
Pigs Weaners North	9	12	18	5	8	13	133	258	509	5	8	13	509	1290	3364
Pigs multipliers North	8	11	17	5	7	12	124	223	453	5	7	12	313	1194	2980
Small Ruminants North	3	8	14	2	4	6	49	87	188	2	4	6	46	126	405
Cattle low trade	4	10	17	3	5	11	96	206	554	3	5	11	76	227	801
Cattle medium trade	6	11	19	3	6	13	145	285	655	3	6	13	144	372	1069

Catle high trade	10	15	22	6	11	21	274	538	907	6	11	21	599	1005	1906
Pigs low trade	9	13	21	5	8	14	217	388	738	5	8	14	319	902	3534
Pigs medium trade	9	13	20	5	8	14	251	427	763	5	9	14	1204	2353	5509
Pigs high trade	11	15	23	8	12	19	387	584	967	9	12	20	4136	6839	13243
Small Rumin high trade	4	10	15	2	4	7	96	174	378	3	4	7	147	303	829
Cattle 2 seeds	13	17	25	12	17	29	572	781	1123	12	17	29	1492	3275	11276
Cattle 3 seeds	14	19	26	16	23	34	682	923	1156	17	23	34	2389	4816	12958
Cattle 4 seeds	15	20	27	19	25	37	725	970	1218	19	25	37	2865	5965	13514
Early detection	6	11	20	3	5	13	128	282	728	3	5	13	98	313	1068
Late detection	7	12	20	3	8	19	149	347	816	3	8	19	152	403	1380
Low resources	6	11	19	3	6	14	108	208	446	3	6	14	129	334	958
High Resources	7	12	20	3	6	15	168	351	973	3	6	15	148	368	1115
StandStill 7 days	6	11	19	3	6	13	134	315	653	3	6	13	134	369	1044
No mov reduc. people	6	11	19	3	6	14	131	274	648	3	6	14	145	338	952
Culling 1Km 1day	5	10	16	3	6	13	126	276	599	6	12	30	292	732	2191
Low Culling resources	5	10	16	3	6	13	125	272	623	6	12	29	260	685	2117
High culling resources	5	10	17	3	6	13	127	277	619	6	13	31	274	709	2279
Culling 0.5km 1day	5	10	17	3	6	14	123	268	632	4	8	20	161	445	1303
Culling 1.5km 1day	4	9	15	3	6	14	118	246	565	9	18	45	403	1095	3400
Culling 1km 7days	4	9	15	3	6	12	115	240	560	5	12	30	263	669	2092
Culling 1km 14days	5	10	16	3	5	13	127	252	598	6	11	28	255	676	2233
Culling 1km 10herds	6	11	18	3	6	14	132	281	637	3	6	19	115	313	1234
Culling 1km 20herds	7	12	19	3	7	15	144	322	728	3	7	15	137	370	1130
Culling 1km 30herds	6	11	19	3	6	13	143	291	681	3	6	13	127	357	1062
Vacc 1km 1day	6	11	18	3	6	14	133	292	698	3	6	13	127	326	962
Low Vcc resources	6	11	19	3	6	14	132	289	693	3	6	14	127	334	1166
High Vacc resources	7	11	20	3	6	15	141	303	729	3	6	15	140	354	1110
Vacc 2km 2day	6	11	19	3	6	14	134	292	692	3	6	14	113	341	1206
Vacc 3km 1 day	6	11	19	3	6	15	142	288	732	3	6	15	130	337	1048
Vacc 1km 7days	6	11	18	3	6	13	138	277	664	3	6	13	133	336	1138
Vacc 1km 14days	6	11	19	3	6	14	131	288	674	3	6	14	124	347	1102
Vacc 1km 10herds	7	12	19	3	6	15	134	301	707	3	6	14	133	387	1222

Vacc 1km 20herds	6	11	19	3	6	14	131	280	652	3	6	14	130	324	1075
Vacc 1km 30herds	6	11	19	3	6	14	135	306	633	3	6	14	134	353	1198
WC-A: low surv. resources	11	15	24	8	12	20	259	363	555	8	12	20	452	872	1818
WC-A: low culling res	11	15	22	8	12	20	368	572	912	8	12	19	473	835	1873
WC-A: detection day28	14	18	25	16	23	40	602	855	1122	16	23	39	998	1738	3571
WC-A: detection day 35	18	22	30	32	50	91	906	1117	1375	32	48	87	2110	3575	7164
WC-A: Standstill -15%	11	15	24	8	12	19	368	560	947	8	12	19	483	858	1936
WC-A: Standstill -40%	11	15	22	8	12	20	373	567	911	8	12	20	468	872	1858
WC-A: Eff. reduction Indir.Cont. -15%	11	15	23	8	12	20	378	582	976	8	12	20	475	930	2075
WC-A: Eff. reduction Indir.Cont. -40%	11	15	24	8	11	21	391	593	983	8	11	21	482	887	1885
WC-A: 3d to trace contacts	11	14	22	7	11	21	360	546	919	7	11	21	472	837	1837
WC-A: Eff. trace Dir.Cont. - 15%	11	15	23	8	12	20	379	570	928	8	12	20	461	868	1856
WC-A: Eff. trace Dir.Cont. - 40%	11	15	22	8	11	22	373	562	937	8	11	22	461	798	2082
WC-A: Eff. trace Indir.Cont. - 15%	11	15	23	8	11	20	390	570	927	8	11	20	483	862	1963
WC-A: Eff. trace Indir.Cont. - 40%	11	15	23	7	12	21	365	564	959	7	12	21	467	815	1793
WC-A: Eff. detect contacts - 15%	11	15	22	7	11	20	385	562	899	7	11	20	464	840	1809
WC-A: Eff. detect contacts - 40%	11	15	23	8	12	21	372	570	935	8	12	21	457	836	1741
WC-A: Eff. control infected -15%	11	15	23	8	12	21	368	568	952	8	12	21	496	905	1924
WC-A: Eff. control infected -40%	11	15	23	8	12	22	383	565	973	8	12	22	461	839	1995
WC-A: Eff. control zones -15%	11	15	23	7	11	21	370	556	986	7	11	21	470	815	1846
WC-A: Eff. control zones -40%	11	15	23	8	12	21	376	592	949	8	12	21	489	941	1888
WC-B: low surv. resources	13	18	26	10	17	31	317	466	621	10	17	31	1178	1624	2616
WC-B: low culling resources	13	18	24	11	17	32	494	755	1088	11	17	32	1199	1671	2688
WC-B: detection day28	17	21	29	30	43	66	873	1089	1294	30	43	65	2322	3278	5031
WC-B: detection day 35	21	25	33	61	88	138	1142	1258	1546	61	85	134	4393	6024	9413
WC-B: Standstill -15%	13	18	25	11	18	33	496	754	1134	11	18	32	1198	1698	2711
WC-B: Standstill -40%	13	18	24	11	18	31	499	758	1075	11	18	31	1228	1684	2738
WC-B: Eff. reduction Indir.Cont. -15%	13	18	26	11	18	30	522	764	1114	11	18	30	1178	1642	2721
WC-B: Eff. reduction Indir.Cont. -40%	13	18	26	11	18	32	505	779	1079	11	18	32	1178	1666	2755
WC-B: 3d to trace contacts	13	17	24	11	18	31	498	738	1081	11	18	31	1182	1631	2618
WC-B: Eff. trace Dir.Cont. - 15%	13	18	25	10	17	31	487	731	1064	10	17	31	1203	1679	2607
WC-B: Eff. trace Dir.Cont. - 40%	13	17	24	11	17	30	500	724	1053	11	17	30	1190	1696	2634
WC-B: Eff. trace Indir.Cont. - 15%	13	17	25	10	17	30	479	729	1073	10	17	30	1191	1677	2666

WC-B: Eff. trace Indir.Cont. - 40%	13	17	24	11	18	32	489	722	1067	11	18	32	1181	1639	2772
WC-B: Eff. detect contacts - 15%	13	17	26	10	18	31	490	750	1096	10	18	30	1190	1659	2590
WC-B: Eff. detect contacts - 40%	13	18	25	10	18	32	483	758	1103	10	18	33	1226	1742	2870
WC-B: Eff. control infected -15%	13	18	26	11	19	33	535	770	1129	11	19	33	1243	1699	2881
WC-B: Eff. control infected -40%	13	18	25	10	17	32	479	739	1082	10	18	32	1191	1712	2763
WC-B: Eff. control zones -15%	13	18	25	10	18	32	501	745	1079	10	18	32	1204	1627	2740
WC-B: Eff. control zones -40%	13	18	25	11	18	31	499	762	1096	11	18	31	1178	1704	2726
WC-C: low surv. resources	15	20	28	20	26	39	443	562	697	20	26	39	3001	6297	13981
WC-C: low culling resources	16	20	26	20	27	39	769	987	1196	20	27	39	2872	6081	14376
WC-C: detection day28	19	24	32	40	54	80	1045	1176	1419	40	53	78	6305	12716	22325
WC-C: detection day 35	24	28	36	87	119	165	1215	1318	1639	87	115	157	14338	23144	35714
WC-C: Standstill -15%	15	19	27	20	27	39	736	976	1222	20	27	39	2852	5488	13431
WC-C: Standstill -40%	15	20	29	20	27	38	744	995	1249	20	28	38	2858	5920	14674
WC-C: Eff. reduction Indir.Cont. -15%	15	20	27	19	27	39	740	992	1232	19	27	39	2739	5804	13660
WC-C: Eff. reduction Indir.Cont. -40%	16	20	28	20	27	39	761	1002	1255	20	27	39	2976	6132	14102
WC-C: 3d to trace contacts	15	20	28	19	27	39	760	987	1235	19	27	39	2874	5553	13694
WC-C: Eff. trace Dir.Cont. - 15%	16	20	29	20	27	39	762	1003	1247	20	27	39	3016	6092	13418
WC-C: Eff. trace Dir.Cont. - 40%	15	20	27	20	26	40	754	982	1211	20	26	39	2898	6096	14360
WC-C: Eff. trace Indir.Cont. - 15%	15	20	28	20	27	40	736	998	1207	20	27	40	2935	6406	14860
WC-C: Eff. trace Indir.Cont. - 40%	15	20	27	20	26	39	746	982	1196	20	26	39	3014	6203	13909
WC-C: Eff. detect contacts - 15%	16	20	29	20	27	39	748	994	1244	20	27	39	3006	6113	14160
WC-C: Eff. detect contacts - 40%	15	20	28	20	26	39	755	978	1230	20	26	39	2751	5623	14195
WC-C: Eff. control infected -15%	15	20	28	20	27	39	740	978	1229	20	27	39	3017	6295	14529
WC-C: Eff. control infected -40%	15	20	27	19	27	38	735	983	1222	19	27	37	2912	5958	13982
WC-C: Eff. control zones -15%	16	20	29	19	27	39	750	1003	1248	19	27	39	2784	6068	13665
WC-C: Eff. control zones -40%	16	21	29	20	27	41	764	1019	1281	20	27	41	2910	6243	14357
Chaos: Base	20	25	33	42	57	83	1075	1194	1439	42	57	82	6682	13095	23002
Chaos: 7d standstill	20	24	33	43	58	85	1081	1188	1440	43	57	83	6787	13481	22925
Chaos: culling	16	19	26	38	50	72	875	1098	1327	86	116	170	12789	19861	29897
Chaos: vaccination	19	22	28	42	57	81	1161	1299	1553	42	56	80	6712	12106	22155

2 Additional figures

Figure S2-1. Results of the scenarios comparing different control measures and amount of resources available. Scenarios are detailed in Figure 2 of the paper. Individual box-plots represent the summary of 1000 iterations for each scenario. Red lines mark the median for all the iterations in the “typical outbreak scenario” against which all measures are compared (first box-plot), and the dashed lines represent the 25% and 75% percentiles for that scenario.

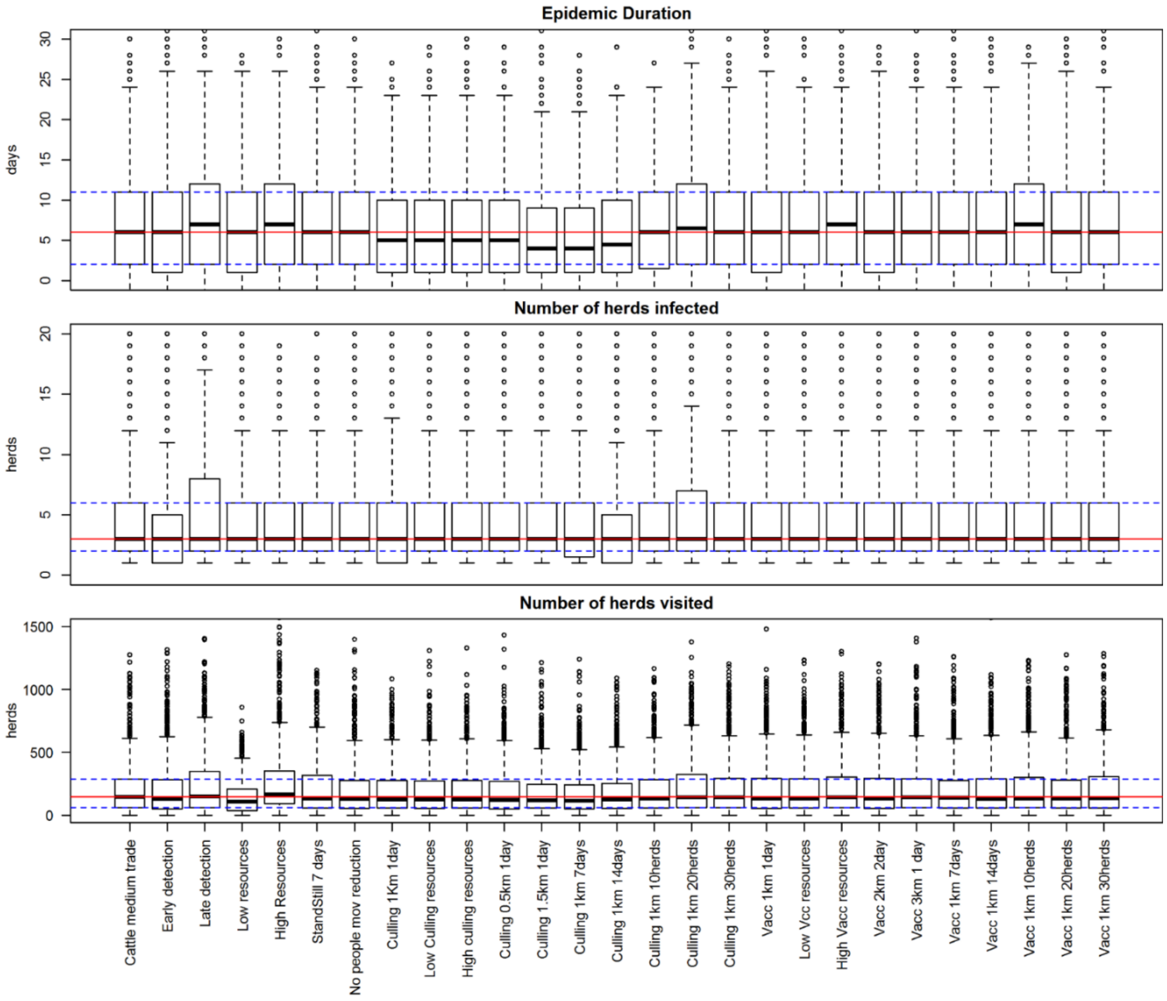

Figure S2-2. Sensitivity analysis for three selected "worst-case scenarios". Scenario are detailed in Table 2 or the paper. Individual box-plots represent the summary of 1000 iterations for each scenario. Red lines mark the median for all the iterations in the "worst-case scenario" against which measures are being compared (first box-plot), and the dashed lines represent the 25% and 75% percentiles for that scenario.

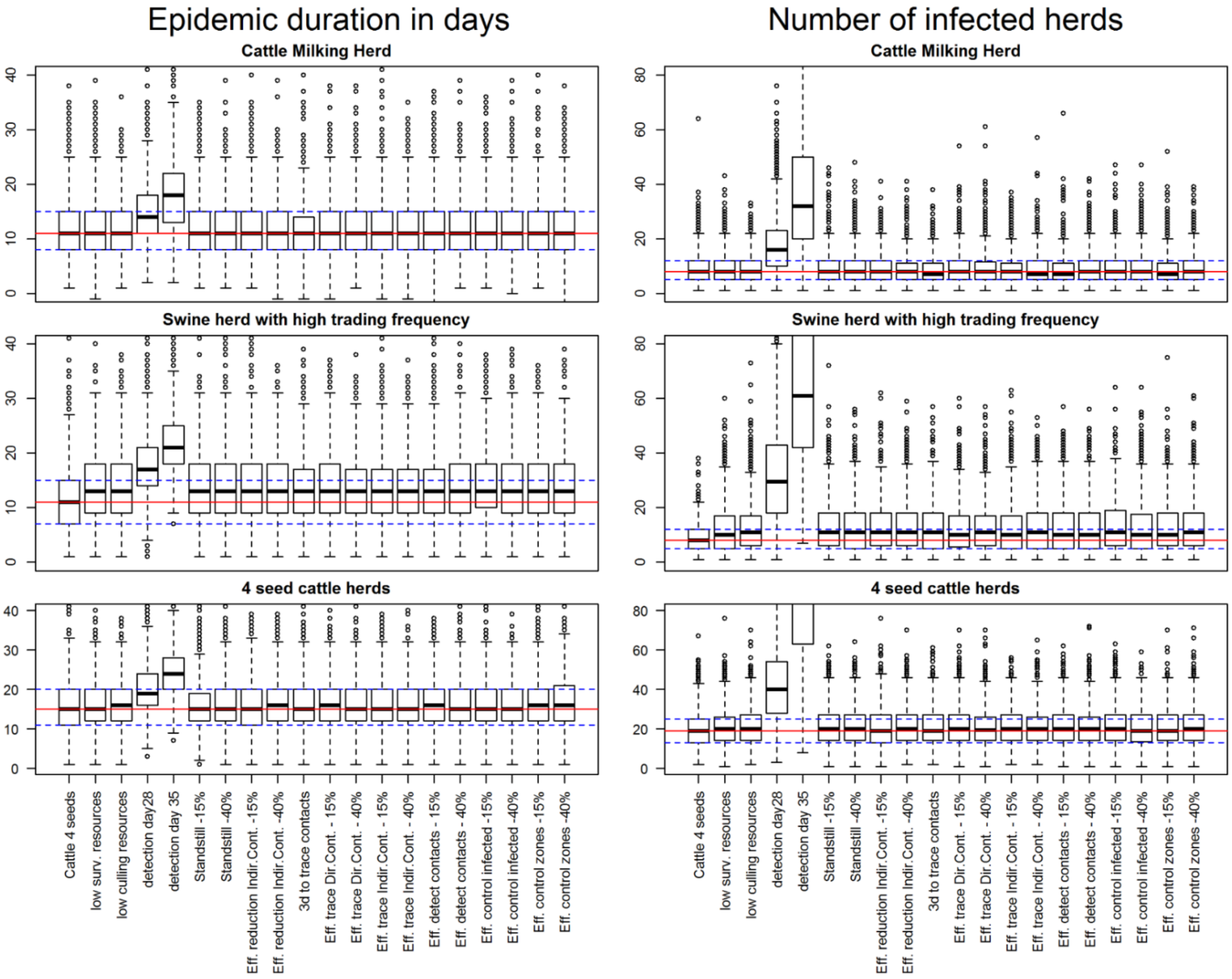

Figure S2-3. Summary statistics for the daily outputs of the “typical outbreak scenario” (ctMid, FMD introduced in a cattle herd with average probability of trading animals), if surveillance capacity is set as 40 surveillance teams per day. Percentiles and maximum of 500 iterations are given. Day 0 is the day the virus is assumed to be introduced in Sweden.

A. Detected herds per day	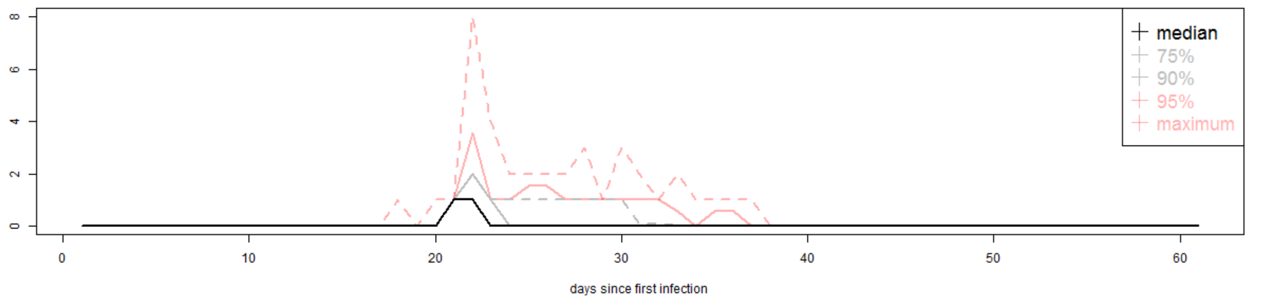
B. Visited herds per day	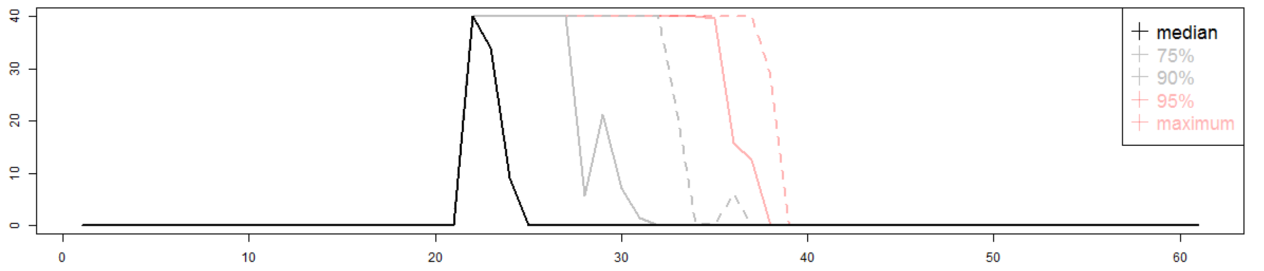
C. Herds waiting to be visited	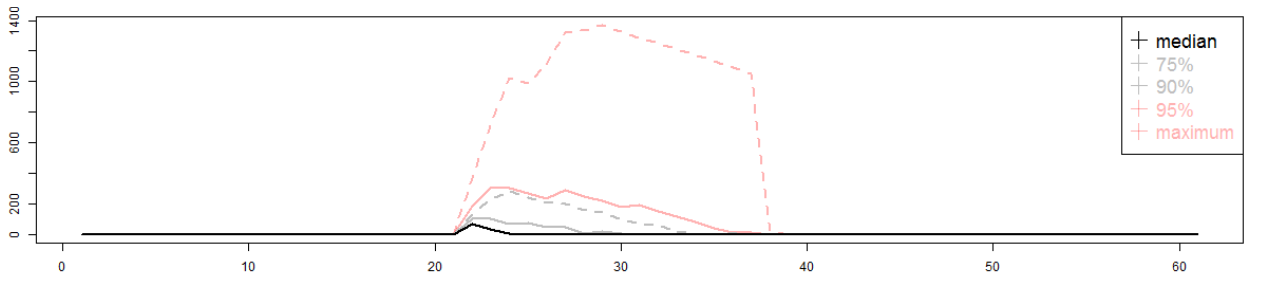

D. Mean number of days waiting to be visited (only accounted for herds in queue, that is, disregarding herds which did not have to wait)	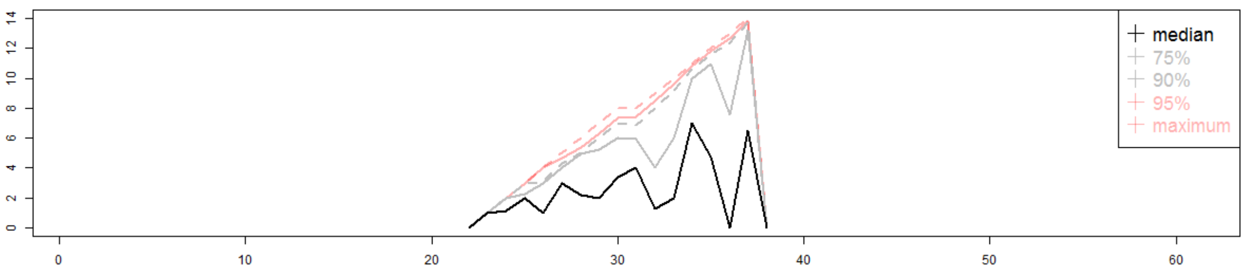
E. Total number of animals infected (to be culled)	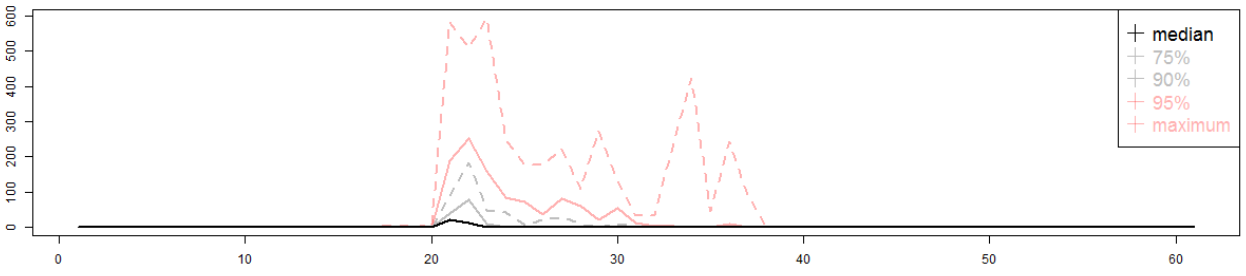
